# Childhood Trauma and Frontoparietal Network Connectivity During Working Memory Task Performance in Individuals With Schizophrenia and Healthy Participants

**DOI:** 10.1111/ejn.70675

**Published:** 2026-09-13

**Authors:** Aoife Warren, David Mothersill, Emma Corley, Derek W. Morris, Brian Hallahan, Declan P. McKernan, Colm McDonald, Gary Donohoe

**Affiliations:** ^1^ School of Psychology and Institute for Health Discovery and Innovation University of Galway Galway Ireland; ^2^ Centre for Neuroimaging, Cognition and Genomics (NICOG), University of Galway Galway Ireland; ^3^ Academic Unit of Neurology, School of Medicine Trinity College Dublin Dublin Ireland; ^4^ School of Medicine University of Galway Galway Ireland; ^5^ School of Biological and Chemical Sciences and Institute for Health Discovery and Innovation University of Galway Galway Ireland; ^6^ Pharmacology & Therapeutics, School of Pharmacy & Medical Sciences University of Galway Galway Ireland

**Keywords:** childhood trauma, frontoparietal network, functional MRI, schizophrenia, working memory

## Abstract

Schizophrenia is associated with altered frontoparietal connectivity, which supports higher‐order cognition, including working memory. Childhood trauma has been linked to altered functional connectivity and reduced cognitive performance in individuals with schizophrenia and controls. Prior evidence suggests that trauma‐related default mode dysconnectivity mediates the association between trauma and cognition. We hypothesised that childhood trauma would be associated with altered frontoparietal connectivity during a working memory task and that such connectivity changes would mediate the relationship between trauma and working memory. Childhood trauma, working memory and fMRI data were collected from individuals with schizophrenia or schizoaffective disorder (*n* = 38) and controls (*n* = 128). fMRI data were preprocessed in CONN, and seed‐based connectivity analyses were performed for four frontoparietal seeds (bilateral dorsolateral prefrontal and posterior parietal cortices). Connectivity was contrasted across (a) diagnosis and (b) trauma severity. Moderated mediation analyses tested the associations between trauma, connectivity and working memory, with diagnosis as a moderator. Across all participants, higher physical neglect severity predicted poorer working memory performance. Stronger inverse connectivity between left dorsolateral prefrontal and frontal medial cortices predicted better working memory performance. As expected, patients showed widespread frontoparietal dysconnectivity relative to controls, but no differences in frontoparietal connectivity were observed across trauma severity groups. Frontoparietal connectivity did not mediate the association between trauma and working memory, although diagnosis moderated both the trauma‐connectivity and connectivity‐cognition associations. We conclude that, unlike previous evidence suggesting a mediating role for the default mode network, frontoparietal connectivity did not mediate the trauma‐cognition association, possibly suggesting the unique significance of default mode network dysconnectivity in linking trauma to cognition in psychosis.

## Graphical abstract

We recorded fMRI data during the N‐back task from 38 individuals with schizophrenia and 128 healthy participants and used moderated mediation analysis to examine the interactions between childhood trauma, frontoparietal connectivity and working memory performance. Connectivity did not mediate the trauma–cognition relationship; however, both the trauma–connectivity and connectivity–cognition associations differed by diagnosis.

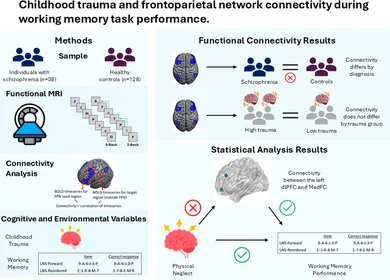

**Abbreviations**
ART: Artifact detection toolbox
BOLD signal: Blood oxygen level dependent
CANTAB: Cambridge Neuropsychological Test Automated Battery
CSF: Cerebrospinal fluid
CT: Childhood trauma
CTQ‐SF: Childhood Trauma Questionnaire–Short Form
dlPFC: Dorsolateral prefrontal cortex
DMN: Default mode network
EA: Emotional abuse
EN: Emotional neglect
FC: Functional connectivity
FD: Framewise displacement
FDR: False detection rate
fMRI: Functional magnetic resonance imaging
FOV: Field of view
FPN: Frontoparietal network
FSIQ: Full‐scale intelligence quotient
GLM: Generalised linear model
HDRS: Hamilton Depression Rating Scale
Hz: Hertz
ICC: Intraclass correlation coefficient
IQ: Intelligent quotient
IQR: Interquartile range
iRelate: ‘Immune Response & Social Cognition in Schizophrenia’ Project
LG: Lingual gyrus
LNS: Letter‐Number Sequencing Task
LP: Lateral parietal cortex
MedFC: Frontal medial cortex
MNI: Montreal Neurological Institute
mPFC: Medial prefrontal cortex
MRI: Magnetic resonance imaging
PA: Physical abuse
PANSS: Positive and Negative Symptom Scale
PFC: Prefrontal cortex
pMTGr: Posterior middle temporal gyrus, right hemisphere
PN: Physical neglect
PPC: Posterior parietal cortex
PrCG: Precentral gyrus
ROI: Region‐of‐interest
SA: Sexual abuse
SBC: Seed‐based connectivity
SCID‐IV: Structured Clinical Interview for the Diagnostic Statistical Manual‐IV
SD: Standard deviation
SPM: Spatial parametric mapping
SWM: Spatial Working Memory
SWMTE: Spatial Working Memory (Total Errors)
SZ: Schizophrenia
SZA: Schizoaffective disorder
WAIS‐III: Wechsler Adult Intelligence Scale—Third Edition

## 1 Introduction

Schizophrenia (SZ) is a neuropsychiatric disorder characterised by disruptions in thought processes, perception and social interaction (Correll and Schooler 2020). Hallucinations, delusions and paranoia are core symptomatology of SZ and are characteristic of psychosis, a transdiagnostic syndrome observed across psychiatric disorders (Häfner 2019). The estimated pooled incidence rate of SZ in Ireland is 20.0 per 100,000 person‐years, representing a substantial proportion of the estimated incidence of all psychotic disorders, which ranges from 22.0 to 25.0 per 100,000 person‐years (Jacinto et al. 2024).

SZ has been robustly associated with widespread alterations in brain structure (Kelly et al. 2018; Lawrie et al. 2008; Rimol et al. 2010; van Erp et al. 2016, 2018) and function (Dong et al. 2018; Lawrie et al. 2008; S. Li et al. 2019; T. Li et al. 2017; Lynall et al. 2010). While genetic influences on structural and functional brain alterations are well established (Lawrie et al. 2008), the role of environmental contributors, including exposure to childhood trauma (CT), is only partly understood. CT is typically assessed in terms of experiences of neglect (physical, emotional) and abuse (physical, emotional and sexual) (Bernstein et al. 1997; Bernstein and Fink 1998). CT is estimated to affect approximately 20%–35% of the general population (Sethi et al. 2013; Stoltenborgh et al. 2013), whereas its prevalence in individuals with SZ is estimated at around 75%–85% (Andrianarisoa et al. 2017; Schalinski et al. 2019; Yousef et al. 2022). Among trauma subtypes, emotional abuse (EA) has the highest self‐reported prevalence rates globally (*M* = 33.6%; 85% CI 30.2%–42.9%), followed by physical abuse (PA) (*M* = 22.6%; 85% CI 20.3%–25.1%) (Stoltenborgh et al. 2015). The prevalence of sexual abuse (SA) is estimated at 18.0% [85% CI 16.9%–19.2%] among females and 7.6% [85% CI 16.9%–19.2%] among males (Stoltenborgh et al. 2015). For neglect, global prevalence rates are estimated at 18.4% [85% CI 14.3%–23.4%] for emotional neglect (EN) and 16.3% [85% CI 13.1%–20.0%] for physical neglect (PN) (Stoltenborgh et al. 2015). Moreover, CT has been linked to increased risk of developing psychosis, with odds ratios varying from approximately 2.8–4.5 across studies (Croft et al. 2019; Heins et al. 2011; McCutcheon et al. 2023; Varese et al. 2012).

### 1.1 Childhood Trauma: Effects on Brain Function and Cognition

The impact of CT on brain development is particularly apparent in stress‐sensitive regions such as the amygdala, hippocampus and prefrontal cortex (PFC) (Danese and McEwen 2012; Mothersill and Donohoe 2016). Reduced amygdala and hippocampal volumes, reduced volumes in the left dorsolateral PFC (dlPFC) and right medial PFC (mPFC) (Danese and McEwen 2012; Driessen et al. 2000; Frodl et al. 2010), and reduced integrity of white matter tracts such as the corpus callosum and corona radiata (McCarthy‐Jones et al. 2018) have each been reported in adults with a history of CT. In addition to structural brain changes, CT has been linked to altered activity and functional connectivity (FC) within the frontoparietal network (FPN) and the default mode network (DMN) (Anticevic et al. 2012, 2013; Dauvermann et al. 2021). FC is defined as the temporal correlation of activity among two or more anatomically distinct regions and can be analysed during rest or during task‐based behaviour (Friston 1994). The FPN is a task‐positive network involved in cognitive control and working memory (Godwin et al. 2017), whereas the DMN is task‐negative and shows most activity at rest or during internally orientated processes such as self‐reflection (Dauvermann et al. 2021). Trauma‐exposed individuals have been reported to show decreased DMN connectivity at rest (Philip et al. 2013a) and greater FPN activation and DMN deactivation during working memory performance (Philip et al. 2016, 2013b) compared to non‐exposed controls.

SZ is associated with deficits in cognitive performance—Individuals with SZ are estimated to perform at 2–3 SD below average for global cognition, and 1–1.5 SD below average for working memory (Kuswanto et al. 2016). These impairments are evident across illness stages—ultra‐high risk, first‐episode and chronic SZ (Aas et al. 2012; McCabe et al. 2012; Üçok et al. 2015)—and they predict poorer psychosocial and functional outcomes in individuals with psychosis (Cowman et al. 2021). CT has also been linked to deficits in general cognitive ability and specific cognitive domains (e.g., memory and social cognition) in individuals with SZ and non‐clinical populations (Aas et al. 2012; Dauvermann and Donohoe 2018). Evidence from a meta‐analysis by Vargas et al. (2019) found modest negative associations between CT and global cognition, and between CT and specific cognitive domains such as working memory and attention. Of note, the CT‐cognition relationship was stronger in control participants than in individuals with psychosis (Vargas et al. 2019).

Changes in FC have been reported to mediate the association between CT and cognition. Specifically, higher CT exposure has been associated with reduced FC within the DMN at rest (Dauvermann et al. 2021). The DMN is also active during internally focused cognitive processes, including self‐referential thinking, autobiographical memory and theory of mind (Dauvermann et al. 2021; Philip et al. 2013a). Higher CT exposure has been associated with reduced connectivity between both the mPFC and precuneus, and between the right lateral parietal cortex (LP) and precuneus, in patients with SZ or schizoaffective disorder (SZA) and healthy participants (Dauvermann et al. 2021). Notably, right LP‐precuneus connectivity partially mediated the relationship between CT and theory of mind performance. Diagnosis was not observed to moderate these associations, suggesting that CT effects on DMN connectivity are comparable across patient and control groups (Dauvermann et al. 2021). Subsequent studies found reduced DMN suppression during social cognitive tasks in SZ/SZA, including between the left LP and the cerebellum (King et al. 2022) and weaker suppression of left LP‐cerebellar connectivity (King et al. 2023).

Evidence that connectivity within the DMN mediates the association between CT and social cognition raises the question of whether other cognitively relevant networks similarly mediate the relationship between CT and cognition. Altered connectivity within the FPN (a brain network underlying processes related to cognitive control) has previously been observed in SZ/SZA and was found to be associated with working memory performance (Eryilmaz et al. 2016; Godwin et al. 2017). Given the high rates of co‐occurrence between SZ and CT exposure, and evidence that CT‐related alterations in DMN connectivity are associated with social cognition, we hypothesised that FPN connectivity during a working memory task might mediate the relationship between CT and working memory performance. Additionally, in line with our previous finding that CT effects on DMN connectivity are not moderated by diagnosis, we hypothesised that the effect of CT on working memory performance via FPN connectivity would not differ significantly between diagnostic groups.

### 1.2 Aims and Hypotheses

The current study investigated whether connectivity within the FPN differs between individuals with low versus high exposure to CT during a working memory task in individuals with SZ/SZA and healthy participants. To address this aim, we conducted seed‐based connectivity (SBC) analyses using four predefined FPN regions‐of‐interest (ROIs): right and left dlPFC, and right and left posterior parietal cortices (PPC). Our analyses focused on FC differences associated with (a) increased working memory load, (b) diagnostic status (schizophrenia vs. controls), (c) low vs. high CT exposure. Based on the evidence above, we hypothesised the following: (1) Individuals with a history of CT would show widespread aberrant FPN connectivity at higher working memory loads; (2) CT‐associated alterations in FPN connectivity would mediate the association between CT and working memory performance; and (3) diagnosis would not moderate the indirect effect of CT on working memory performance via FPN connectivity (i.e., the mediation effect of FPN connectivity on the trauma‐cognition association would be comparable across patient and control groups).

## 2 Materials and Methods

### 2.1 Study Participants

One hundred and ninety individuals from the ‘Immune Response & Social Cognition in Schizophrenia’ (iRelate) research project were included in this study. Forty‐three individuals with SZ or SZA were recruited through community mental health services in Dublin and Galway, Ireland. Patients were aged 18–65, had a chronic illness history and were diagnosed with SZ/SZA as confirmed using the Structured Clinical Interview for the Diagnostic and Statistical Manual of Mental Disorders, Fourth Edition (SCID‐IV). All participants were clinically stable outpatients at the time of assessment, as per the opinion of their treatment team. Exclusion criteria included any history of neurological disorders (e.g., epilepsy) or head injury with loss of consciousness for greater than 1‐min, comorbid Axis I mental health disorders, an estimated IQ of less than 70, substance use disorder within the past month, reported pregnancy/lactation and any MRI contraindications (metal implants, claustrophobia).

One hundred and forty‐seven healthy participants were recruited via media advertising in Galway and Dublin. Controls met the same general eligibility criteria as patients and, in addition, had no personal history of Axis I mental illness, no first‐degree relative with a psychotic disorder and no self‐reported substance abuse in the 6 months before recruitment.

All participants provided written informed consent under protocols approved by the local Ethics Committees of the Galway University Hospitals, National University of Ireland Galway and Tallaght Hospital (ethics number for Galway = CA1441).

Five patients and 19 controls were excluded from the analysis arising from scanner artefacts (*n* = 3), >10 outlier scans for motion (*n* = 9), field of view limitations (*n* = 7) or missing log files (*n* = 5) (see Supplementary Table S1). This resulted in a sample size of *N* = 166 (patients: *n* = 38; controls: *n* = 128) for inclusion in the statistical analysis.

### 2.2 Childhood Trauma History

CT was retrospectively assessed using the Childhood Trauma Questionnaire (CTQ) – Short Form (Bernstein et al. 1997, 2003; Bernstein and Fink 1998). The CTQ is a self‐report measure of experiences of early life adversity comprising 5 subscales of physical abuse (PA), physical neglect (PN), emotional abuse (EA), emotional neglect (EN) and sexual abuse (SA). Each subscale contains five items rated on how frequently the event was experienced during childhood using a Likert scale from 1 (‘never true’) to 5 (‘very often true’). There was no missing CTQ data for the 166 included participants; total CTQ scores and scores for the five trauma subscales were available for all participants.

The CTQ‐SF shows good internal consistency (Cronbach's α = 0.891), and excellent test–retest reliability (intraclass correlation coefficient (ICC) = 0.788) across clinical and non‐clinical populations (Badenes‐Ribera et al. 2024). Specifically, good internal consistency (Cronbach's α = 0.874) and excellent test–retest reliability (ICC = 0.772–0.81) have been noted for the CTQ‐SF among individuals with SZ (Jiang et al. 2018; Xiang et al. 2021).

### 2.3 Neuropsychological Assessment

Working memory was assessed using the Letter‐Number Sequencing (LNS) and Spatial Working Memory (SWM) tests. LNS is a supplemental subtest of the WAIS‐III which requires examinees to listen to a sequence of numbers and letters and recall the numbers in ascending order and the letters in alphabetical order (Wechsler 1997b, 1997a). The stimuli begin with two alphanumeric characters per string and gradually increase in difficulty until the stimuli comprise eight characters per string. There are seven string lengths (or trials) with three items per trial. Fully correct responses are awarded one point each, and possible scores range from 0 to 21 (Mielicki et al. 2018). The reliability estimate (average reliability coefficient, calculated via Fisher z transformation) for the LNS subtest of the WAIS‐III is r_xx_ = 0.82 (Silva 2008; Wechsler 1997b).

The SWM from the CANTAB (Sahakian and Owen 1992) requires examinees to find yellow ‘tokens’ hidden among coloured boxes while avoiding boxes that they have already searched. Total errors (SWMTE) were used as the measure of working memory performance in this case, whereby fewer errors indicate better working memory performance. SWMTE is the combined sum of SWM‐between errors (revisiting boxes that have already been found to contain a token) and SWM‐within errors (revisiting boxes that have already been found to be empty) (Kumar et al. 2016) (see Appendix 1 for further details). SWMTE shows moderate test–retest reliability (Pearson's *r* = 0.68) (Lowe and Rabbitt 1998).

To describe the general cognitive ability of participants, full‐scale IQ (FSIQ) was estimated using three subtests from the Wechsler Adult Intelligence Scale version III (WAIS‐III): Vocabulary, Digit Symbol and Block Design (Wechsler 1997a). Several two‐subtest and four‐subtest short forms for FSIQ have been validated (Grégoire and Wierzbicki 2009; Ringe et al. 2002), of which a frequently used two‐subtest version (comprised of Vocabulary and Block Design subtests) correlates highly with the full‐scale WAIS‐III (*r =* 0.92) (Axelrod 2002; Grégoire and Wierzbicki 2009). In the present study, we included an additional subtest in our FSIQ measure—Digit Symbol—to capture attentional processes which are often impaired in SZ (Dichter et al. 2010). Our group previously found that this three‐subtest version correlated highly with the validated two‐subtest measure (Corley et al. 2024). Reliability estimates (average reliability coefficient, calculated via Fisher *z* transformation) for the constituent subtests are good to excellent: Vocabulary showed excellent internal consistency (r_xx_ = 0.93), while Digit Symbol (r_xx_ = 0.84) and Block Design (r_xx_ = 0.86) showed good internal consistency (Silva 2008; Wechsler 1997b).

### 2.4 Neuroimaging Data Acquisition

Brain imaging was acquired using a 3 Tesla Philips Achieva MR system (Philips Medical Systems, Best, The Netherlands) with gradient strength 80 mT/m and slew rate 200 T/m/s using an 8‐channel receive‐only head coil at the Centre for Advanced Medical Imaging, St. James's Hospital, Dublin, Ireland.

#### 2.4.1 Structural MRI

T1‐weighted brain images were acquired using a 3D Inversion Recovery Spoiled Gradient Recalled echo (IR‐SPGR) sequence with: FOV = 256 × 256 × 160 mm^3^, spatial resolution = 1 mm^3^, TR/TE = 8.5/3.9 ms, TI = 1060 ms, flip angle = 8°, SENSE factor = 1.5, acquisition time = 7 min 30 s. T2‐weighted images were acquired using a turbo spin echo (TSE) sequence with turbo factor 15 and with: FOV = 230 × 184 × 149 mm^3^, spatial resolution = 0.57 × 0.72 × 4 mm^3^, 30 slices with 1 mm gap, TR/TE = 3000/80 ms, with no SENSE parallel imaging employed, acquisition time = 1 min 48 s.

#### 2.4.2 Task‐Based Functional MRI

Task‐based fMRI was acquired using single‐shot echo planar imaging (SS‐EPI) and a dynamic scan time per scan of 2 s with: FOV = 224 × 224 × 122.85 mm^3^, spatial resolution = 2.8 mm^3^, 39 slices with 0.349999905 mm interslice gap, TR/TE = 2000/35 ms with SPIR fat suppression. The acquisition time was 7 min 28 s, with a total of 224 volumes acquired. The first 4 volumes were classed as dummy scans and were excluded on this basis, resulting in a total of 220 volumes being retained for further analysis.

Participants completed the N‐back task (2 conditions: 0‐back and 2‐back) in the scanner during image acquisition. The two conditions were analysed separately to identify connectivity patterns associated with low cognitive load (0‐back) and high cognitive load (2‐back). Furthermore, a conditions contrast (2‐back > 0‐back) was applied to isolate brain connectivity patterns associated with increasing cognitive load while controlling for activity present during the lower load condition (see Appendix 2).

### 2.5 Neuroimaging Data Analysis

Analyses of fMRI data were performed using CONN (RRID: SCR_009550) release 22.a (Whitfield‐Gabrieli and Nieto‐Castanon 2012) and SPM (RRID: SCR_007037) release 12.7771 (Friston et al. 2007). Full details of the neuroimaging data analysis (including preprocessing, denoising, first‐ and group‐level analyses) are outlined in Appendix 3.

#### 2.5.1 Preprocessing

Functional and anatomical data were preprocessed using a modular preprocessing pipeline including realignment, slice timing correction, outlier detection, direct segmentation, MNI‐space normalisation and smoothing.

#### 2.5.2 Denoising

The artefact detection toolbox (ART) was used to identify scans showing excessive head motion. ‘Motion scrubbing’ was applied to limit the effects of head movement. Regressors denoting ‘outlier’ scans (10 components), motion parameters and their first‐order derivatives (12 factors), white matter and cerebrospinal fluid (CSF) time series (16 CompCor noise components each), and linear trends (2 factors) were included in the general linear model (GLM), and the BOLD time series was band‐pass filtered (0.01–0.1 Hz).

Participants with more than 10 outlier scans were excluded from the analysis (*N* = 9; patients: *n* = 4, controls: *n* = 5). Motion parameters were compared across patient and control groups, and high‐ and low‐trauma groups. Patients had significantly larger mean framewise displacement (FD) (patients: *M* = 0.261, *SD* ± 0.146; controls: *M* = 0.160, *SD* ± 0.067; *t*_(39.35)_ = 3.854, *p* < 0.001), and significantly greater outlier counts (patients: median = 2, IQR = 7.25; controls: median = 0, IQR = 2; *W* = 1581, *p* = 0.003) than controls. There was no significant difference in mean FD across low‐ and high‐trauma groups (low‐trauma: *M* = 0.173, *SD* ± 0.101; high‐trauma: *M* = 0.197, *SD* ± 0.095; *t*_(121.93)_ = −1.533, *p* = 0.128). However, the high‐trauma group had greater outlier counts compared to the low‐trauma group (low‐trauma: median = 0, IQR = 2; high‐trauma: median = 2, IQR = 5; *W* = 2237.5, *p* = 0.006) (Supplementary Table S2A,B).

#### 2.5.3 Functional Connectivity Analysis

SBC analyses were conducted for four predefined FPN seeds: left dlPFC (−43, 33, 28), left PPC (−46, −58, 49), right dlPFC (41, 38, 30) and right PPC (52, −52, 45). FC was evaluated using multivariate regression coefficients from a weighted‐GLM. SBC was analysed for the 0‐back and 2‐back conditions separately, and a 2‐back > 0‐back contrast was also applied to isolate connectivity patterns associated with increasing cognitive load.

Between‐group analyses were conducted for each condition, and connectivity was compared across (a) patients vs. controls and (b) high CT vs. low CT groups. The between‐groups analysis for high vs. low CT groups was conducted to broadly explore the effects of trauma severity on FPN connectivity, as previously employed in studies by Philip and colleagues (Philip et al. 2016, 2013a, 2013b). Participants were dichotomised into high‐ and low‐trauma groups using a mean split cross‐validated by the severity classification thresholds from the CTQ manual (Bernstein and Fink 1998), which was conducted for total CTQ scores only. A mean split was preferred over a median split as the sample mean was 37.0, which aligns closely with the none/minimal versus low‐moderate threshold in the CTQ manual (≤ 36). Given the relatively low levels of trauma in our sample, we considered this an appropriate cut‐off, which is both clinically grounded and sample‐representative. Individuals with a total CTQ score of ≤ 36 were assigned to the ‘low‐trauma’ group (*n* = 103, *M* = 29.53, *SD* ± 3.18) and the remaining participants were assigned to the ‘high‐trauma’ group (*n* = 57, *M* = 51.35, *SD* ±13.85).

Age and sex were included as covariates for all between‐group analyses, given their well‐established associations with functional connectivity and cognitive performance (Murman 2015; C. Zhang et al. 2016; S. Zhang et al. 2025).

Seed‐to‐voxel regression analyses were performed to assess linear associations between SBC of the four FPN seeds and trauma severity (indexed by PN or total CTQ scores as continuous regressors). These associations were tested for the 0‐back and 2‐back conditions separately, and for the 2‐back > 0‐back contrast. Age and sex were included as covariates in all regression analyses.

For all SBC analyses, inferences used parametric statistics from Gaussian random field theory with a *p* < 0.001 voxel‐level threshold, and a *p*‐FDR < 0.05 cluster‐size threshold. All SBC analysis results were visualised using MRIcroGL (version 1.2.20220720; Rorden 2025).

### 2.6 Statistical Analysis of Demographic and Cognitive Variables

All statistical analyses were performed in RStudio v2024.12.0.467 (Posit team 2025) using R Statistical Software v4.1.2 (R Core Team 2025).

Group comparisons for patients and healthy participants were conducted for demographic and cognitive variables using chi‐square (*χ* ^2^) tests for sex and handedness, and two‐tailed independent samples *t*‐tests for all other variables. In cases where education history (calculated as total years of post‐primary education) was missing, the values were imputed from IQ (*n* = 6), using a simple imputation method in RStudio using the mice package (Buuren and Groothuis‐Oudshoorn 2011).

### 2.7 Linear Regression Analyses

#### 2.7.1 Preliminary Regressions

Preliminary linear regressions between CT scores (including all 5 subscales and the total score) and working memory measures (LNS total score and SWMTE) were conducted in RStudio using the stats package (R Core Team 2025). These tested for direct associations between trauma exposure and working memory performance. Sex, age and education history were included as covariates in all regression analyses given their well‐reported effects on working memory performance (Bloomberg et al. 2021; Boller et al. 2017; Fukuda et al. 2010). Bonferroni correction was applied to adjust for multiple testing where appropriate.

#### 2.7.2 Linear Regressions Between Trauma, Connectivity and Working Memory

Linear regressions were carried out in RStudio using the stats package (R Core Team 2025) to assess the associations between (a) CT and FPN connectivity and (b) FPN connectivity and working memory, while controlling for sex, age and education history. Bonferroni correction was applied to adjust for multiple comparisons.

### 2.8 Moderated Mediation Analyses

Moderated mediation models (PROCESS macro model 59) were carried out using the processR package (Moon 2023) to test whether the relationship between CT (X) and working memory (Y) was mediated by FPN connectivity (M), and whether diagnosis (W) moderated these associations. Age, sex and education history were included as covariates in the analysis, given their well‐documented effects on cognition (Bloomberg et al. 2021; Boller et al. 2017; Fukuda et al. 2010).

## 3 Results

### 3.1 Demographic, Clinical and Environmental Data

Demographic, clinical and environmental variables are presented in Table 1. No significant difference was observed between patients and controls for sex, but differences were noted for age (patients: *M* = 41.7, *SD* ±10.6; controls: *M* = 35.4, *SD* ±12.1; *t*_(68.11)_ = 3.14, *p* = 0.003), education history (patients: *M* = 15.1, *SD* ± 2.8; controls: *M* = 16.8, *SD* ± 4.0; *t*_(75.47)_ = −3.03, *p* = 0.0032) and IQ (patients: *M* = 95.8, *SD* ±17.0; controls: *M* = 114.6, *SD* ±16.6; *t*_(59.38)_ = −5.98, *p* < 0.001). Hamilton Depression Rating Scale (HDRS) scores indicated low depressive symptomatology across all participants (patients: *M* = 3.45, *SD* ±3.75; controls: *M* = 3.03, *SD* ± 3.66). In patients, Positive and Negative Symptom Scale (PANSS) total scores were consistent with expectations for a clinically stable outpatient group (*M* = 39.16, *SD* ± 9.01).

**TABLE 1:** Means and group differences for demographic, clinical, environmental and cognitive variables.

Characteristics	Patients (*N* = 38)	Healthy participants (*N* = 128)	Statistics	
	Mean (SD)	Mean (SD)	*t*/*χ* ^2^	*p*
Gender (male/female)	29:9	74:54	*χ* ^2^ = −3.511	0.061
Age (years)	41.7 (10.6)	35.4 (12.1)	*t*_*(*68.11)_ = 3.140	0.002**
Handedness^a^	34:3	112:13	*χ* ^2^ = −0.009	0.923
Years of education^b^	15.1 (2.8)	16.8 (4.0)	*t*_(75.47)_ = −2.905	0.005**
Total CTQ score	41.8 (16.7)	35.6 (12.0)	*t*_(48.86)_ = 2.138	0.038*
Emotional abuse	9.9 (5.3)	8.4 (4.2)	*t*_(51.46)_ = 1.613	0.113
Physical abuse	7.0 (4.0)	6.4 (2.7)	*t*_(47.40)_ = 0.8581	0.395
Sexual abuse	7.1 (5.2)	5.5 (1.6)	*t*_(39.11)_ = 1.900	0.065
Physical neglect	7.5 (3.4)	6.3 (2.3)	*t*_(47.79)_ = 2.082	0.0428*
Emotional neglect	10.4 (4.9)	9.1 (4.3)	*t*_(55.15)_ = 1.4575	0.1507
Total WASI IQ	95.8 (17.0)	114.6 (16.6)	*t*_(59.38)_ = −5.979	<0.001***
LNS (total score)	8.3 (3.3)	11.6 (2.3)	*t*_(48.35)_ = −5.950	<0.001***
SWMTE	16.8 (8.7)	8.2 (8.4)	*t*_(59.27)_ = 5.375	<0.001***
Diagnosis (SZ:SZA)	29:9			
Duration of illness (years)^c^	17.8 (10.4)			
HDRS symptom severity	3.45 (3.75)	3.03 (3.66)	*t*_(59.40)_ = 0.6036	0.5484
PANSS total score^d^	39.16 (9.01)			
PANSS positive symptoms^d^	8.57 (2.22)			
PANSS negative symptoms^d^	10.35 (4.22)			
PANSS general symptoms^d^	20.51 (4.25)			
Primary antipsychotic medication^e^	(a) 3; (b) 8; (c) 2; (d) 11; (e) 1; (f) 1; (g) 1; (h) 2			
Additional antipsychotic medication^f^	(a) 1; (b) 1; (c) 1; (d) 1; (e) 1; (f) 1			
Other medication^g^	(a) 12; (b) 1; (c) 4			

*Note:* Two‐tailed independent *t*‐tests were conducted to assess group differences for all variables with the exception of sex and handedness which were evaluated using chi‐square tests. Handedness recorded as ratio of right‐handed individuals to left‐handed individuals; CTQ = Childhood Trauma Questionnaire; WASI = Wechsler Abbreviated Scale of Intelligence; IQ = Intelligence Quotient; LNS = Letter‐Number Sequencing test from Wechsler Adult Intelligence Scale III; SWMTE = Spatial Working Memory Total Errors from the CANTAB; SZ = Schizophrenia; SZA = Schizoaffective Disorder; HDRS = Hamilton Depression Rating Scale; PANSS = Positive and Negative Syndrome Scale.

Abbreviations: CTQ, Childhood Trauma Questionnaire; HDRS, Hamilton Depression Rating Scale; IQ, intelligence quotient; LNS, Letter‐Number Sequencing test from Wechsler Adult Intelligence Scale III; PANSS, Positive and Negative Syndrome Scale; SZA, schizoaffective disorder; SZ, schizophrenia; SWMTE, Spatial Working Memory Total Errors from the CANTAB; WASI, Wechsler Abbreviated Scale of Intelligence.

^a^ Missing data for handedness: HC (N) = 3, 1 ambidextrous individual in patient group.

^b^ Missing data for years of education: HC (N) = 3, SZ (N) = 3.

^c^ Missing data for duration of illness: SZ (N) = 2.

^d^ Missing data for PANSS total, positive, negative, general: SZ (N) = 1 in each case.

^e^ (a) Apriprazole, (b) clozapine, (c) fluphenazine, (d) olanzapine, (e) paliperidone, (f) quetiapine, (g) risperidone, (h) clopoxil.

^f^ (a) Apriprazole, (b) clopoxil, (c) paliperidone, (d) quetiapine, (e) amisulpride, (f) ziprasidone.

^g^ (a) Antidepressant, (b) benzodiazepine, (c) mood stabiliser.

* *p* < 0.05.

** *p* < 0.01.

*** *p* < 0.001.

Patients showed significantly higher total CTQ (patients: *M* = 41.8, *SD* ± 16.7; controls: *M* = 35.6, *SD* ± 12.0; *t*_(48.86)_ = 2.14, *p* = 0.038) and PN (patients: *M* = 7.5, *SD* ± 3.4; controls: *M* = 6.3, *SD* ± 2.3; *t*_(47.79)_ = 2.08, *p* = 0.043) scores compared to controls. No other CTQ subscales showed group differences. Total CTQ and PN scores both positively correlated with PANSS‐positive scores (total CTQ, *r* = 0.41, *p* = 0.012; PN, *r* = 0.34, *p* = 0.039) and PANSS‐total scores (total CTQ, *r* = 0.34, *p* = 0.039; PN, *r* = 0.35, *p* = 0.036) but not with PANSS‐negative scores (total CTQ, *r* = 0.21, *p* = 0.211; PN, *r* = 0.26, *p* = 0.121) or PANSS‐general scores (total CTQ, *r* = 0.24, *p* = 0.145; PN, *r* = 0.25, *p* = 0.139).

No significant difference was observed between high‐ and low‐trauma groups for sex or education history. However, individuals in the high‐trauma group were significantly older than individuals in the low‐trauma group (low‐trauma group: *M* = 35.0, *SD* ± 11.7; high‐trauma group: *M* = 40.3, *SD* ± 12.1; *t*_(110.59)_ = −2.74, *p* = 0.007) (see Supplementary Table S3).

### 3.2 Childhood Trauma and Working Memory Performance

As expected, patients performed significantly worse on both LNS (patients, *M* = 8.3, *SD* ± 3.3; controls, *M* = 11.6, *SD* ± 2.3; *t*_(48.35)_ = −5.95, *p* < 0.001) and SWMTE (patients, mean = 16.8, *SD* ±8.7; controls, *M* = 8.2, *SD* ± 8.4; *t*_(59.27)_ = 5.38, *p* < 0.001) compared to controls.

In preliminary regression analyses, *higher* PN scores were associated with *poorer* LNS performance (*B* = −0.26, *SE* = 0.08, *p* = 0.002, adjusted *p* value = 0.012), indicating that per unit increase in PN, LNS scores decreased by 0.26 units. A significant nominal association was noted between *higher* PN and *higher* SWMTE (*B* = 0.50, *SE* = 0.25, *p* = 0.044, adjusted *p* value = 0.265). A significant nominal association was also noted for *higher* physical abuse scores and *poorer* LNS performance (*B* = −0.18, *SE* = 0.07, *p* = 0.014, adjusted *p* value = 0.083) (see Supplementary Table S4 and Supplementary Figures S1 and S2). However, only the PN‐LNS association survived Bonferroni correction (adjusted *p* value = 0.012). Hence, this association was brought forward for further testing as the direct pathway between trauma and cognition for the moderated mediation stage.

### 3.3 Seed‐Based Connectivity of the Fronto‐Parietal Network (2‐Back vs. 0‐Back Contrast)

The high vs. low working memory load (2‐back > 0‐back) contrast was associated with significant differences in FC for two FPN seed regions: the left dlPFC (13 associations) and right dlPFC (1 association). There were no significant differences in FC for either PPC seed region for this contrast (see Supplementary Table S5 and Supplementary Figure S3 for full SBC results).

Based on their statistical significance, three associations were brought forward to the linear regression stage: (1) left dlPFC and medial frontal cortex (MedFC), (2) left dlPFC and left lingual gyrus (LG) and (3) right dlPFC and posterior middle temporal gyrus, right hemisphere (pMTGr).

### 3.4 Seed‐Based Connectivity of the Fronto‐Parietal Network (Controls vs. Patients)

#### 3.4.1 Group Differences (Patients vs. Controls) During the 2‐Back Condition

**FIGURE 1:**
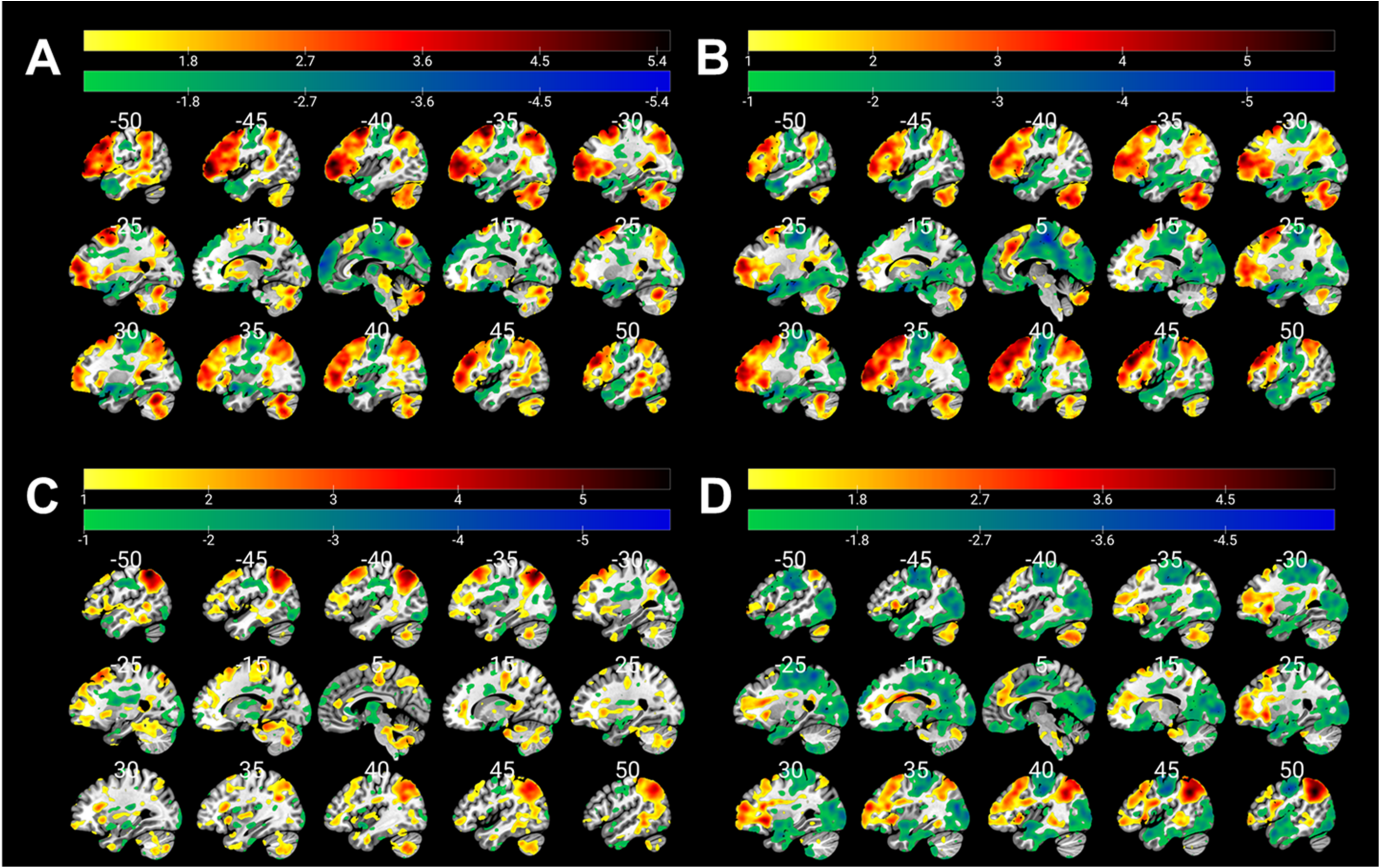
Heat maps of seed‐based connectivity (SBC) results for frontoparietal seed regions during the 2‐back condition, showing differences between healthy participants and individuals with schizophrenia (SZ/SZA). (A) Left dorsolateral prefrontal cortex (dlPFC) seed: nine clusters showed significantly increased (yellow‐red) or decreased (green‐blue) connectivity in SZ/SZA compared to healthy participants. (B) Right dlPFC seed: thirteen clusters showed significant connectivity alterations. (C) Left posterior parietal cortex (PPC) seed: two clusters showed significant connectivity alterations. (D) Right PPC seed: three clusters showed significant connectivity alterations.

During the 2‐back condition, patients showed widespread altered connectivity compared to healthy participants across all FPN seed regions: left dlPFC (9 associations), right dlPFC (13 associations), left PPC (2 associations) and right PPC (3 associations) (Figure 1; see Table 2).

**TABLE 2:** Significant seed‐based associations showing group differences for controls > patients between frontoparietal network seed regions and target voxels brain‐wide during the 2‐back condition.

Seed region	MNI space (*x*, *y*, *z*)	Target region	MNI space (*x*, *y*, *z*)	Voxels	Peak T	Height threshold PFDR value
Left dlPFC	(−43, 33, 28)	Frontal pole left	(−48, 50, 04)	2321	5.5818	<0.0001***
Left dlPFC	(−43, 33, 28)	Middle frontal gyrus left	(−40, 10, 58)	913	5.5387	<0.0001***
Left dlPFC	(−43, 33, 28)	Frontal pole left	(−08, 62, 30)	739	−4.7476	<0.0001***
Left dlPFC	(−43, 33, 28)	Cingulate gyrus, anterior division	(00, −12, 34)	494	−5.1313	0.0003***
Left dlPFC	(−43, 33, 28)	Middle frontal gyrus right	(46, 34, 24)	378	4.9024	0.0004***
Left dlPFC	(−43, 33, 28)	Superior parietal lobule left	(−36, −56, 50)	459	4.6397	0.0016**
Left dlPFC	(−43, 33, 28)	Middle frontal gyrus right	(34, 18, 60)	299	5.0931	0.0036**
Left dlPFC	(−43, 33, 28)	Frontal pole right	(42, 44, 04)	275	4.5548	0.0048**
Left dlPFC	(−43, 33, 28)	Cerebellum Crus I right	(34, −60, −32)	220	4.9237	0.0117*
Right dlPFC	(41, 38, 30)	Frontal pole right	(46, 38, 28)	3267	5.597	<0.0001***
Right dlPFC	(41, 38, 30)	Precentral gyrus right	(02, −28, 50)	1506	−5.4066	<0.0001***
Right dlPFC	(41, 38, 30)	Frontal pole left	(−28, 58, 16)	1334	5.1217	<0.0001***
Right dlPFC	(41, 38, 30)	Temporal pole left	(−22, 12, −30)	640	−4.7049	<0.0001***
Right dlPFC	(41, 38, 30)	Postcentral gyrus right	(50, −14, 44)	490	−4.4965	0.0002***
Right dlPFC	(41, 38, 30)	Cerebellum 7b left	(−38, −58, −46)	429	4.9349	0.0004***
Right dlPFC	(41, 38, 30)	Frontal orbital cortex right	(22, 12, −22)	411	−5.5818	0.0005***
Right dlPFC	(41, 38, 30)	Middle frontal gyrus left	(−40, 12, 60)	279	4.8043	0.0037**
Right dlPFC	(41, 38, 30)	Planum temporale right	(62, −10, 02)	253	−4.2131	0.0053**
Right dlPFC	(41, 38, 30)	Precentral gyrus left	(−22, −26, 60)	191	−4.3211	0.0156*
Right dlPFC	(41, 38, 30)	Cingulate gyrus, posterior division	(−06, −42, 06)	187	−4.0335	0.0156*
Right dlPFC	(41, 38, 30)	Paracingulate gyrus right	(08, 26, 38)	181	4.4098	0.0162*
Right dlPFC	(41, 38, 30)	Left hippocampus	(−22, −22, −14)	177	−5.0636	0.0162*
Left PPC	(−46, −58, 49)	Angular gyrus left	(−50, −52, 50)	1405	5.6832	<0.0001***
Left PPC	(−46, −58, 49)	Lateral occipital cortex, superior division right	(48, −60, 44)	195	4.1332	0.0171*
Right PPC	(52, −52, 45)	Angular gyrus right	(50, −50, 40)	1197	5.296	<0.0001***
Right PPC	(52, −52, 45)	Postcentral gyrus right	(58, −12, 46)	349	−5.1655	0.0048**
Right PPC	(52, −52, 45)	Occipital pole right	(00, −90, 02)	269	−4.0751	0.0123*

Abbreviations: dlPFC, dorsolateral prefrontal cortex; PPC, posterior parietal cortex.

* *p* < 0.05.

** *p* < 0.01.

*** *p* < 0.001.

#### 3.4.2 Group Differences (Patients vs. Controls) During the 0‐Back Condition

During the 0‐back condition, patients showed widespread alterations compared to healthy participants in connectivity for all predefined FPN seeds: left dlPFC (7 associations), right dlPFC (9 associations), left PPC (4 associations) and right PPC (3 associations) (see Supplementary Table S6 and Supplementary Figure S4 for full SBC results).

#### 3.4.3 Group Differences (Patients vs. Controls) for 2‐Back > 0‐Back Contrast

**FIGURE 2:**
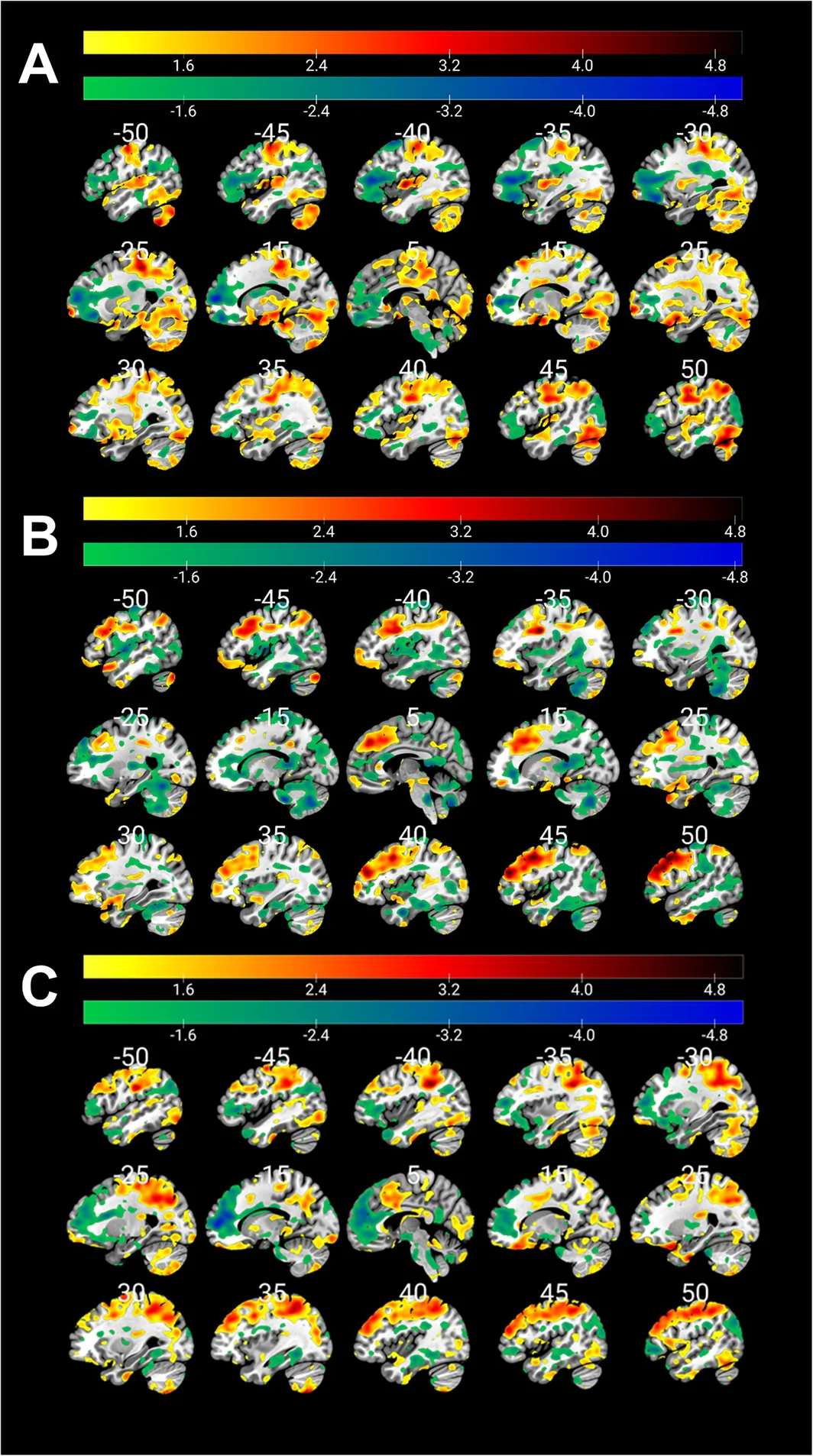
Heat maps of seed‐based connectivity (SBC) results for frontoparietal seed regions under increasing working memory load (2‐back > 0‐back contrast), showing differences between healthy participants and individuals with schizophrenia (SZ/SZA). (A) Right dorsolateral prefrontal cortex (dlPFC) seed: four clusters showed significantly increased (yellow‐red) or decreased (green‐blue) connectivity in SZ/SZA compared to healthy participants. (B) Left posterior parietal cortex (PPC) seed: one cluster showed significantly reduced connectivity in SZ/SZA compared to healthy participants. (C) Right PPC seed: two clusters showed significant connectivity alterations.

Under increasing working memory load (2‐back > 0‐back contrast), we observed significant differences in FPN connectivity between patients and controls for the following FPN seeds: right dlPFC (4 associations), left PPC (1 association) and right PPC (2 associations) (Figure 2). No significant connectivity differences between patients and controls were detected for the left dlPFC seed (see Table 3).

**TABLE 3:** Significant seed‐based associations showing group differences for controls > patients between frontoparietal network seed regions and target voxels brain‐wide under increasing WM load (2‐back > 0‐back contrast).

Seed region	MNI space (*x*, *y*, *z*)	Target region	MNI space (*x*, *y*, *z*)	Voxels	Peak T	Height threshold PFDR value
Right dlPFC	(41, 38, 30)	Left frontal pole	(−16, 58, 08)	181	4.7284	0.0421*
Right dlPFC	(41, 38, 30)	Left frontal pole	(−36, 38, 08)	178	4.3669	0.0421*
Right dlPFC	(41, 38, 30)	Right precentral gyrus	(58, −04, 32)	165	−4.2267	0.0421*
Right dlPFC	(41, 38, 30)	Right cerebellum (1)	(50, −58, −26)	163	−5.0111	0.0421*
Left PPC	(−46, −58, 49)	Right middle frontal gyrus	(52, 24, 28)	525	−4.6014	0.0002***
Right PPC	(52, −52, 45)	Left paracingulate gyrus	(−12, 54, 10)	435	5.0709	0.0011**
Right PPC	(52, −52, 45)	Right postcentral gyrus	(60, −12, 48)	182	−4.9913	0.0425*

Abbreviations: dlPFC, dorsolateral prefrontal cortex; PPC, posterior parietal cortex.

* *p* < 0.05.

** *p* < 0.01.

*** *p* < 0.001.

### 3.5 Seed‐Based Connectivity of the Fronto‐Parietal Network (Low CT vs. High CT Contrast)

#### 3.5.1 Group Differences (Low > High Trauma) During the 2‐Back Condition

During the 2‐back condition, there were no significant connectivity differences between high vs. low trauma groups for any of the FPN seeds across all participants. When analysing patients and controls separately, no differences in FPN connectivity between high vs. low trauma subgroups were detected during the 2‐back condition.

#### 3.5.2 Group Differences (Low > High Trauma) During the 0‐Back Condition

During the 0‐back condition, there were no significant differences in FPN connectivity between high vs. low trauma groups across the whole sample. Furthermore, no differences in connectivity during the 0‐back were found between high vs. low trauma subgroups when the patient and control groups were analysed separately.

#### 3.5.3 Group Differences (Low > High Trauma) for 2‐Back > 0‐Back Contrast

Across all participants, no significant connectivity differences were observed between high vs. low CT groups for any of the FPN seed regions. In patients only, when contrasting low vs. high working memory load, the *high* CT subgroup showed *reduced* connectivity between the left dlPFC and left cerebellum compared to the low CT subgroup (MNI coordinates: [−46, −70, −26], cluster‐size = 329 voxels, peak T = −6.0304, *p*FDR = 0.0007). In controls only, no significant differences in connectivity were detected between high vs. low CT groups.

### 3.6 Functional Connectivity of the FPN and Childhood Trauma

#### 3.6.1 Seed‐to‐Voxel Regression Analyses Between Trauma and FPN Connectivity During 2‐Back

Seed‐to‐voxel regression analyses were conducted in CONN to test the associations between FPN connectivity during the 2‐back condition and total CTQ or PN scores. The regression analyses indicated that higher total CTQ scores were associated with stronger connectivity between the left dlPFC and a cluster encompassing the left precentral gyrus (PrCG) (peak MNI coordinates: [36, −12, 46], cluster‐size = 293 voxels, peak T = 4.5719, *p*FDR = 0.0120). No other significant associations between trauma scores and FPN connectivity were detected during the 2‐back condition.

#### 3.6.2 Seed‐to‐Voxel Regression Analyses Between Trauma and FPN Connectivity During 0‐Back

Seed‐to‐voxel regression analyses were performed in CONN to test the associations between trauma severity and FPN connectivity during the 0‐back condition. No significant associations were detected between trauma scores (total CTQ or PN) and FPN connectivity during the 0‐back condition.

#### 3.6.3 Seed‐to‐Voxel Regression Analyses Between Trauma and FPN Connectivity for the 2‐Back > 0‐Back

Seed‐to‐voxel regression analyses were conducted to investigate the relationship between trauma scores and changes in FPN connectivity associated with increasing working memory load. The regression analyses indicated that higher PN scores were associated with reduced connectivity of the left PPC and a cluster covering the right cerebellum, lobule VIII (peak MNI coordinates: [14, −70, −34], cluster‐size = 156 voxels, peak T = −5.3484, *p*FDR = 0.0306). No other significant associations were observed between trauma scores and FPN connectivity under increasing cognitive load.

### 3.7 Linear Regression Analyses Between Trauma and FPN Connectivity

Linear regressions were used to test for associations between PN and the selected FC measures (left dlPFC‐MedFC, left dlPFC‐left LG, right dlPFC‐pMTGr). No significant associations were observed between PN and the FC measures of interest (see Supplementary Table S7, Supplementary Figure S5).

### 3.8 Linear Regression Analyses Between FPN Connectivity and Working Memory

Linear regressions between the selected FC measures and working memory performance showed a significant association between connectivity of the left dlPFC and MedFC and LNS performance, whereby s*tronger* negative connectivity predicted *better* LNS performance (*B* = −3.22, *SE* = 1.29, *p* = 0.014, adjusted *p* value = 0.042). Specifically, a one‐unit decrease in FC value—corresponding to increased dlPFC activity and reduced MedFC activity—was associated with a 3.22‐point increase in LNS score. Sex, age and years of education were included as covariates and Bonferroni correction was applied. No other significant associations were noted between FC and SWMTE or LNS scores for our sample (see Supplementary Table S8, Supplementary Figure S6). Following these findings, connectivity between the left dlPFC and MedFC was brought forward to serve as the mediator in the moderated mediation analysis.

### 3.9 Moderated‐Mediation Analysis

**FIGURE 3:**
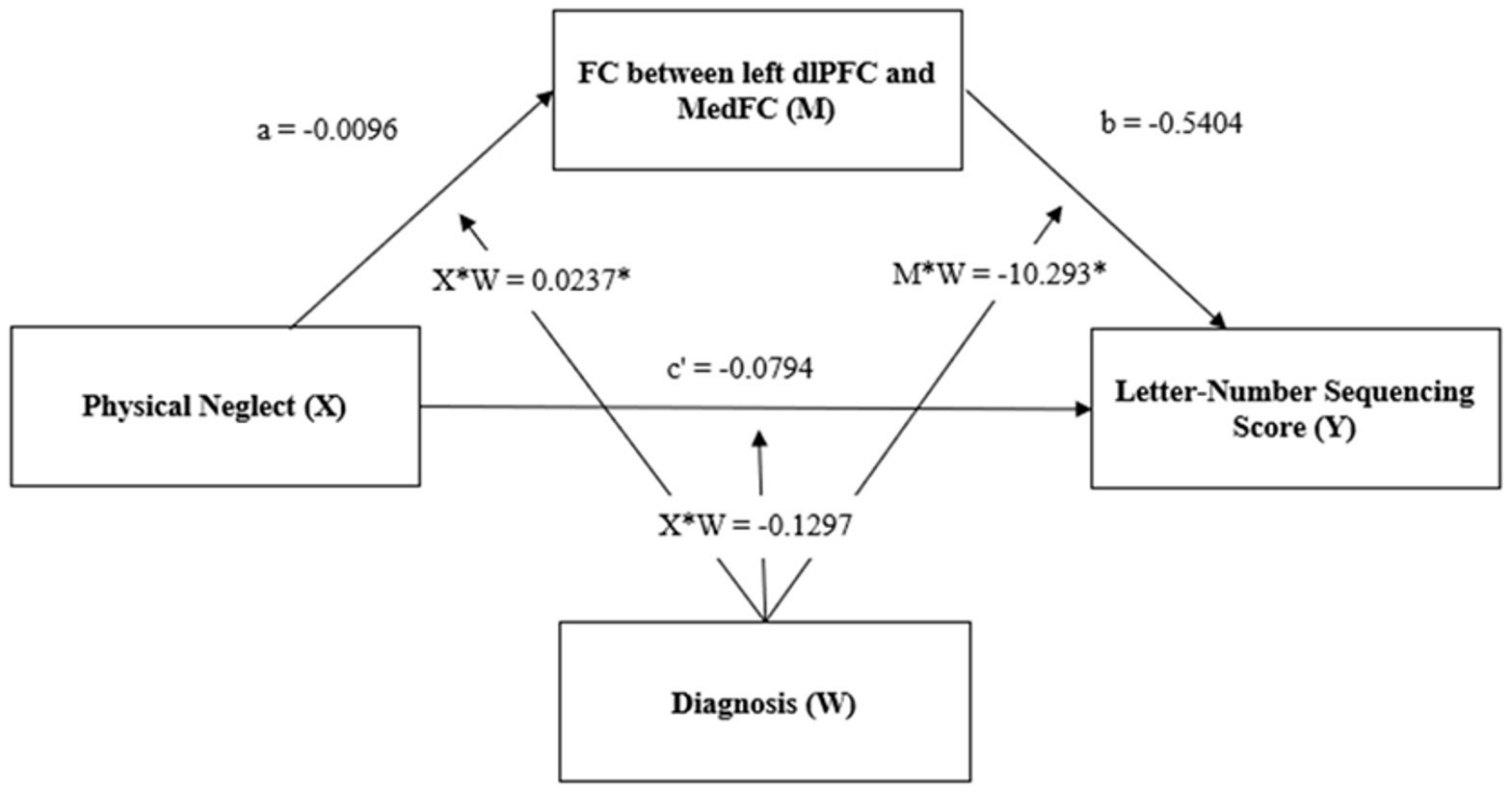
Moderated‐mediation path analysis diagram including connectivity between the left dorsolateral prefrontal cortex (dlPFC) and the frontal medial cortex (MedFC) as the mediator. There was a significant index of moderated mediation for the model including connectivity between the left dlPFC and the MedFC as the mediator. The moderator (diagnosis) affected (a) the effect of physical neglect on connectivity between the left dlPFC and the MedFC (*R* ^2^ change = 2.98%, *p* = 0.0223), and (b) the effect of left dlPFC‐MedFC connectivity on LNS performance (*R* ^2^ change = 2.75%, *p* = 0.0112). Both associations were found to be stronger in patients than in control subjects.

Moderated mediation analysis was conducted with PN serving as the predictor (X), LNS scores as the outcome (Y), connectivity between the left dlPFC and MedFC as the mediator (M) and diagnosis as the moderator (W) variables. Sex, age and education history were included as covariates. There was no evidence that connectivity between the left dlPFC and the MedFC mediated the relationship between PN and LNS performance. However, the index of moderated mediation was significant (*index* = −0.1582, BootSE = 0.1064, 95% CI: [−0.4141, −0.008]), suggesting that the magnitude of the indirect effect is 0.158 points lower in patients than in controls. Diagnosis explained an additional 2.98% of variance in the PN–FC association (*R* ^2^ change = 2.98%, *p* = 0.0223) and an additional 2.75% in the FC–LNS relationship (*R* ^2^ change = 2.75%, *p* = 0.0112), suggesting that the associations were moderated by diagnosis. Despite this moderation effect, the PN–FC association was not significant within controls (*b* = −0.0096, *p* = 0.1326) or patients (*b* = 0.0141, *p* = 0.0828) when examined separately. The FC–LNS relationship reached significance only among patients (*b* = −10.83, *p* = 0.0054), whereby a one‐unit decrease in FC value—corresponding to increased dlPFC activity and reduced MedFC activity—was accompanied by a 10.83 increase in LNS scores (Figure 3; Supplementary Table S9).

## 4 Discussion

### 4.1 Summary of Key Findings

This study examined the effects of CT on FPN connectivity during a working memory task in individuals with SZ/SZA and healthy participants and examined whether such effects are related to cognitive performance. Whole‐group seed‐based analyses indicated no differences in FPN connectivity between high‐ and low‐CT groups during the 2‐back or 0‐back conditions, nor under increasing cognitive load (2‐back > 0‐back contrast). Among patients only, the high‐CT subgroup displayed reduced connectivity between the left dlPFC and left cerebellum (crus I) under increasing working memory load.

Seed‐to‐voxel regression analyses indicated that higher PN scores were associated with reduced connectivity between the left PPC and right cerebellum (lobule VIII) under increasing load, and that higher total CTQ scores were linked to stronger connectivity between the left dlPFC and right PrCG during the 2‐back.

We also observed that stronger negative connectivity of the left dlPFC and the MedFC was associated with better LNS performance across all participants. However, this connectivity index did not mediate the PN–LNS relationship. Both the PN–FC and FC–LNS relationships differed by diagnosis; however, given the discrepancy in sample size between patient (*n* = 38) and control (*n* = 128) groups, these findings should be interpreted with caution.

### 4.2 Childhood Trauma, Functional Connectivity and Cognitive Performance

In contrast to our hypotheses, we found that trauma severity (high vs. low trauma exposure) was not associated with altered FPN connectivity, and that trauma‐related changes in FPN connectivity did not mediate the trauma‐cognition association. Our results are inconsistent with a recent study from Huang et al. (2026) who found that dynamic connectivity of the FPN mediated the association between total trauma and accuracy on the N‐back task. These discrepancies may reflect the differences in working memory tests (working memory capacity vs. updating measures) and connectivity measures (static vs. dynamic) employed across studies. During the LNS, attention processes support the maintenance of relevant information, whereas for the N‐back, attention control supports updating of the target and disposal of outdated information (Burgoyne et al. 2025). Our static connectivity measure correlated seed and target region activity separately during the 0‐back and 2‐back conditions and contrasted them using a 2‐back > 0‐back comparison. The approach taken in Huang et al. (2026) looks at individual‐level modulation of FPN connectivity over time in response to increasing working memory load. The temporal variability in FPN connectivity induced by increased cognitive load during the N‐back task may be more sensitive to trauma effects than static measures of FPN connectivity.

The present findings contrast with evidence from previous fMRI studies by our group (Dauvermann et al. 2021; King et al. 2022, 2023), and others (Cancel et al. 2017), that report an association between trauma exposure, CT‐related alterations in DMN connectivity, and social cognitive performance. Using data from the sample employed in the present study, our group has previously shown that trauma exposure is linked to hypoactivity of the DMN at rest and hyperactivity of the DMN during a social cognitive task. In both instances, the trauma‐related connectivity changes were found to mediate the trauma‐cognition association, and this effect did not differ between patient and control groups. The discrepancies between this prior evidence and the findings of the present study are twofold. Firstly, trauma‐related changes in FPN connectivity did not mediate the trauma–cognition relationship, and secondly, diagnosis moderated the associations between CT, FPN connectivity, and working memory performance. Taken together, these inconsistencies may serve to further underline the significance of trauma‐linked dysconnectivity of the DMN in relation to reduced cognitive performance in trauma‐exposed individuals.

Although not hypothesised, we found a correlation between higher total CTQ scores and *stronger* connectivity between the left dlPFC and right PrCG. We also found evidence for CT‐related alterations in prefrontal‐cerebellar connectivity during working memory performance. While the PrCG and the cerebellum are typically associated with motor coordination, both regions have previously been found to be activated during working memory tasks (Brissenden et al. 2021; Emch et al. 2019; Tan et al. 2025). The PrCG has previously been observed to be similarly activated in both trauma‐exposed individuals and non‐exposed controls during the N‐back task (Philip et al. 2016). Furthermore, prior evidence from our group suggested that trauma‐exposed individuals with SZ/SZA exhibit DMN‐cerebellar dysconnectivity (Dauvermann et al. 2021; King et al. 2022) and, further to this, the significance of prefrontal–cerebellar dysconnectivity during working memory performance in SZ/SZA has been highlighted by prior neuroimaging studies (Meyer‐Lindenberg et al. 2005; Orban et al. 2017).

Taken together, these findings may reflect network‐ and/or task‐specific effects of trauma on brain connectivity. The DMN, which supports self‐referential and affective processes (Buckner 2013), appears more vulnerable to CT‐related changes than the FPN, a central cognitive control network (Godwin et al. 2017), which seems comparatively resilient. This view is supported by evidence from neuroimaging studies investigating resilience to stress, which indicates that FPN function is flexible, adaptive and relatively resilient to functional disruption following trauma exposure (Roeckner et al. 2021). Comparatively, the DMN may be more susceptible to trauma effects and shows early maladaptive changes to stress exposure (Norbury et al. 2023; Roeckner et al. 2021).

### 4.3 Childhood Trauma and Working Memory Performance

On a behavioural level, we found that higher PN exposure predicted worse LNS performance. Contrary to our hypotheses, this trauma‐cognition association was not mediated by CT‐related changes in FPN connectivity, suggesting that the pathways linking trauma to working memory performance deficits may be relatively independent of FPN connectivity. In contrast, we found widespread case–control differences in connectivity for all four FPN seed regions. FPN dysconnectivity in SZ possibly represents a trait‐like feature of this condition, rather than a consequence of CT exposure. For example, these connectivity changes possibly reflect the effects of alternative factors that contribute to psychosis risk, such as neurodevelopmental (Fatemi and Folsom 2009), genetic (Cattarinussi et al. 2022), or neurobiological (Cross et al. 2017) vulnerabilities, dopamine dysfunction (Abi‐Dargham et al. 2002) or gene–environment interactions (Wahbeh and Avramopoulos 2021).

### 4.4 Limitations and Suggestions for Future Studies

Our findings should be interpreted in light of a number of limitations. First, despite the large sample size (*n* = 166), our final sample included only 38 patients. While this is not of major concern for whole‐group analyses examining the effects of trauma, when examining case–control differences, the small patient sample reduces statistical power and limits the robustness of these between‐group inferences. Second, the cross‐sectional design precludes conclusions about causality and restricts the interpretation of confounding variables such as antipsychotic use, which is known to impact brain connectivity (Zeng et al. 2022). Third, the use of the Childhood Trauma Questionnaire, which is both subjective and retrospective, introduces potential recall and reporting biases arising from the natural decay of memory and differences in personality types and coping styles. Lastly, although it was outside the scope of this study to model other potential moderators of CT effects, such as genetic susceptibilities (Caspi et al. 2003), developmental timing (Andersen and Teicher 2008), and trauma type or multiplicity (De Bellis and Zisk 2014; Teicher et al. 2006), it is likely that modelling such factors would have provided additional insights into the nature of trauma effects on brain connectivity.

Future research could employ novel modelling and machine learning approaches to longitudinal, multimodal data to investigate the temporal relationships between trauma exposure, brain functional connectivity, and cognitive and clinical outcomes. For example, longitudinal mediation models, cross‐lagged panel models and causal machine learning approaches could be employed to explore and clarify causal pathways linking childhood trauma, brain function and cognitive dysfunction in psychosis.

## 5 Conclusion

To our knowledge, this is the first study to examine the effects of CT exposure on frontoparietal connectivity during a working memory task in healthy controls and individuals with SZ/SZA and to explore the mediating role of the frontoparietal network in the relationship between CT and working memory performance. The limited evidence observed here for an association between CT and FPN connectivity, taken alongside previous evidence linking trauma‐related alterations in the DMN to social cognitive deficits, suggests that CT exposure may have effects that are pathway‐specific, rather than having more general effects. If confirmed by future studies, such findings may guide the development of interventions targeting CT‐associated cognitive dysfunction by suggesting which specific pathways (e.g., the DMN) to target.

## Author Contributions

**Aoife Warren:** conceptualisation, methodology, investigation, formal analysis, visualisation, resources, writing — original draft, writing — review and editing. **David Mothersill:** conceptualisation, methodology, writing — review and editing, supervision. **Emma Corley:** writing — review and editing. **Derek W. Morris:** writing — review and editing. **Brian Hallahan:** writing — review and editing. **Declan P. McKernan:** writing — review and editing. **Colm McDonald:** writing — review and editing. **Gary Donohoe:** conceptualisation, methodology, formal analysis, investigation, writing — original draft, writing — review and editing, funding acquisition, project administration.

## Funding

This work was supported by the European Research Council, 10.13039/501100000781, ERC‐2015‐STG‐677467; the Health Research Board, 10.13039/100010414, RL‐2020‐007; and the Science Foundation Ireland, 10.13039/501100001602, SFI‐16/ERCS/3787.

## Ethics Statement

All participants provided informed consent prior to inclusion in the study as per the ethics approval pertaining to the study provided by Galway University Hospital (CA1441).

## Conflicts of Interest

The authors declare no conflicts of interest.

## Code Availability

The codes supporting the findings of this study are available from the first author (AW) upon reasonable request.

## Supporting information


**Table S1:** Participant exclusion data including the numbers of the whole sample, controls and patients excluded on the basis of the different data exclusion criteria.
**Figure S1:** Scatterplots showing the associations between childhood trauma (CT) scores (total CTQ and all 5 subscales) and working memory performance as measured by the letter‐number sequencing (LNS) test. The panels below show the associations between: (a) physical neglect and LNS scores (B = −0.26, p = 0.0019**, p‐adj = 0.0116*), (b) emotional neglect and LNS scores (B = −0.01, p = 0.8601, p‐adj = 1), (c) physical abuse and LNS scores (B = −0.18, p = 0.0138*, p‐adj = 0.0827), (d) emotional abuse and LNS scores (B = −0.08, p = 0.1185, p‐adj = 0.7109), (e) sexual abuse and LNS scores (B = −0.04, p = 0.0695, p‐adj = 1), (f) total CT and LNS scores (B = −0.03, p = 0.0598, p‐adj = 0.3589).
**Figure S2:** Scatterplots showing the associations between childhood trauma (CT) scores (total CTQ and all 5 subscales) and working memory performance as measured by the spatial working memory total errors (SWMTE). The panels below show the associations between: (a) physical neglect and SWMTE (B = 0.50, p = 0.0442*, p‐adj = 0.2652), (b) emotional neglect and SWMTE (B = 0.2811, p = 0.0562, p‐adj = 0.337), (c) physical abuse and SWMTE (B = 0.37, p = 0.0804, p‐adj = 0.4823), (d) emotional abuse and SWMTE (B = 0.14, p = 0.3417, p‐adj = 1), (e) sexual abuse and SWMTE (B = 0.06, p = 0.7999, p‐adj = 1), (f) total CT and SWMTE (B = 0.0893, p = 0.0695, p‐adj = 0.4172).
**Figure S3:** Heat maps of seed‐based connectivity (SBC) results for frontoparietal seed regions showing significant differences in connectivity under increasing working memory (WM) load (2‐back > 0‐back contrast). (A) Left dorsolateral prefrontal cortex (dlPFC) seed: 13 clusters showed significantly increased (yellow‐red) or decreased (green‐blue) connectivity under increasing WM load. (B) Right dlPFC seed: one cluster showed significantly reduced connectivity under increasing WM load.
**Figure S4:** Heat maps of seed‐based connectivity (SBC) results for frontoparietal seed regions showing significant differences in connectivity between healthy participants and individuals with schizophrenia during the 0‐back condition. (A) Right dorsolateral prefrontal cortex (dlPFC) seed: seven clusters showed significantly increased (yellow‐red) or decreased (green‐blue) connectivity in SZ when compared to healthy participants. (B) Left dlPFC seed: nine clusters showed significantly increased or decreased connectivity in SZ compared to healthy participants. (C) Right posterior parietal cortex (PPC) seed: four clusters showed significantly increased or decreased connectivity in SZ compared to healthy participants. (D) Left PPC seed: three clusters showed significantly increased or decreased connectivity in SZ compared to healthy participants.
**Figure S5:** Scatterplots showing the associations between physical neglect (PN) scores and frontoparietal connectivity. The panels below show the associations between: (a) PN and connectivity between the left dorsolateral prefrontal cortex (dlPFC) and frontal medial cortex (MedFC) (*B* = 0.0015, p = 0.7619, p‐adj = 1); (b) PN and connectivity between the right dlPFC and the middle temporal gyrus, posterior division (pMTG) (B = 0.0019, p = 0.6675, p‐adj = 1); (c) PN and connectivity between the left dlPFC and left lingual gyrus (LG) (B = −0.0036, p = 0.3685, p‐adj = 1).
**Figure S6:** Scatterplots showing the associations between frontoparietal connectivity and letter number sequency (LNS) scores. The panels below show the associations between: (a) connectivity of the left dorsolateral prefrontal cortex (dlPFC) and frontal medial cortex (MedFC) and LNS scores (B = −3.22, p = 0.0139*, p‐adj = 0.0416*); (b) connectivity of the right dlPFC and the middle temporal gyrus, posterior division (pMTG) and LNS scores (B = −1.624, p = 0.2791, p‐adj = 0.8372); (c) connectivity of the left dlPFC and left lingual gyrus (LG) (B = −2.746, p = 0.1025, p‐adj = 0.3076).
**Table S2A:** Means and group differences for mean framewise displacement and outlier count for patients and healthy participants.
**Table S2B:** Means and group differences for mean framewise displacement and outlier count for low‐ and high‐trauma groups.
**Table S3:** Means and group differences for sex, age and years of education for low‐ and high‐trauma groups.
**Table S4:** Regression results for childhood trauma questionnaire (CTQ) total and subscale scores and a) letter‐number sequencing (LNS) scores and b) spatial working memory total errors (SWMTE).
**Table S5:** Significant seed‐based associations between frontoparietal network seed regions and target voxels brain‐wide for the 2‐Back ‾ 0‐back contrast across the whole sample.
**Table S6:** Significant seed‐based associations showing group differences for controls ‾ patients between frontoparietal network seed regions and target voxels brain‐wide during the 0‐back condition.
**Table S7:** Regression results for physical neglect and the seed‐based connectivity measures between a) the right dlpfc and right pMTG, b) Left dlPFC and MedFC, c) Left dlPFC and Left LG.
**Table S8:** Regression results for the seed‐based connectivity measures between a) the right dlPFC and right pMTG, b) left dlPFC and MedFC, c) left dlPFC and left LG, and letter‐number sequencing scores.
**Table S9:** Moderated mediation (PROCESS Macro Model 59) results for the model with connectivity between the left dorsolateral prefrontal cortex and the frontal medial cortex (MedFC) as the mediator.
**Table S10:** Zero‐Order Correlation matrix between childhood trauma scores (all 5 subscales and total trauma scores), working memory performance (letter‐number sequencing scores, and spatial working memory total errors), and frontoparietal connectivity (left dorsolateral prefrontal cortex (dlPFC) and frontal medial cortex (MedFC); left dlpfc and left lingual gyrus (LG); Right dlPFC and the middle temporal gyrus, posterior division (pMTG)).

## Data Availability

The data that support the findings of this study are available from the corresponding author (GD) upon reasonable request.
