# Supplementary material for: Childhood Trauma and Frontoparietal Network Connectivity During Working Memory Task Performance in Individuals With Schizophrenia and Healthy Participants
Source: Eur J Neurosci. 2026 Sep 13;64(5):e70675. doi: 10.1111/ejn.70675 (PMC13572810; doi:10.1111/ejn.70675)
Supplement: Supplementary file 1 [file EJN-64-0-s001.docx]

**Supplementary Table 1.**

| **Exclusion Criterion** | **Controls (*n*)** | **Patients (*n*)** | **Total (*N*)** |
| --- | --- | --- | --- |
| Scanner artifacts | 3 | 0 | 3 |
| Missing log file | 5 | 0 | 5 |
| Outlier scans^a^ | 5 | 4 | 9 |
| Field of view issues | 6 | 1 | 7 |
| **Total** | 19 | 5 | 24 |

*Participant Exclusion Data Including the Numbers of the Whole Sample, Controls and Patients Excluded on the Basis of the Different Data Exclusion Criteria*

*Note.* In total, 24 participants were excluded (controls, *n* = 19; patients, *n* = 5), resulting in a sample size of *N* = 166 (patients: *n* = 38; controls: *n* = 128) for inclusion in the statistical analysis.

^a^Individuals with >10 outlier scans for excessive head motion were excluded.

**Appendix 1**

*Assessing Working Memory Performance*

Letter-Number Sequencing (LNS) is a supplemental subtest of the Wechsler Adult Intelligence Scale III (WAIS-III) (Wechsler, 1997) in which the examinee is read a sequence of numbers and letters and is then asked to recall the numbers in ascending order and the letters in alphabetical order. It assesses the working memory (WM) component of verbal IQ. The LNS requires several cognitive abilities including attention, concentration, mental manipulation, sequential processing, memory span and short-term auditory memory. For this study, the LNS subtest comprised of 7 trials with three items per trial and hence, the range of possible scores spanned from 0-21.

The Spatial Working Memory (SWM) subtest of the CANTAB (Sahakian & Owen, 1992) requires the examinee to attempt to find yellow ‘tokens’ among a number of coloured square boxes on a computer screen. The examinee must click on each box in turn until a box reveals a yellow token. They must then continue this process until the empty column on the right-side of the screen is filled with yellow tokens. Clicking on a box which was previously shown not to contain a token or revisiting a box that had already been shown to contain a token is counted as an error. For our sample, the outcome of interest for the SWM test was total errors (SWMTE).

**Appendix 2**

*The N-Back Task*

In the current study, the N-Back task was comprised of two conditions with varying difficulties – the 0-Back (control) condition and the 2-Back (task) condition. During the 0-Back condition, the participant was asked to press a button when a specified target appeared on the screen. The 0-Back requires the participant to maintain the target in WM either for the task duration, or until the target is changed, drawing primarily on identification and maintenance processes. During the 2-Back condition, the participant was asked to press a button if the stimulus on screen matched the stimulus shown two trials back. As with the 0-Back condition, the 2-Back draws from identification and maintenance processes, however, it additionally requires updating and inhibition of distractors as the participant must maintain an additional distractor stimulus in WM and inhibit a response if matched on the next trial. Contrasting higher-load conditions (e.g., 2-Back) with lower-load conditions (e.g., 0-Back) can be used to isolate brain connectivity patterns that are associated with increased difficulty (or load) of the WM task.

**Appendix 3**

The details of the neuroimaging analysis as conducted in CONN (RRID:SCR_009550) release 22.a and SPM (RRID:SCR_007037) release 12.7771 are detailed below.

**Preprocessing:** Functional and anatomical data were preprocessed using a modular preprocessing pipeline (Nieto-Castanon, 2020) including realignment with correction of susceptibility distortion interactions, slice timing correction, outlier detection, direct segmentation and MNI-space normalization, and smoothing. Functional data were realigned using SPM realign & unwarp procedure (Andersson, Hutton, Ashburner, Turner, & Friston, 2001), where all scans were coregistered to a reference image (first scan of the first session) using a least squares approach and a 6 parameter (rigid body) transformation (Friston et al., 1995), and resampled using b-spline interpolation to correct for motion and magnetic susceptibility interactions. Temporal misalignment between different slices of the functional data (acquired in ascending order) was corrected following SPM slice-timing correction (STC) procedure (Henson, Buechel, Josephs, & Friston, 1999; Sladky et al., 2011), using sinc temporal interpolation to resample each slice BOLD timeseries to a common mid-acquisition time. Potential outlier scans were identified using ART (Whitfield-Gabrieli, Nieto-Castanon, & Ghosh, 2011) as acquisitions with framewise displacement above 0.9 mm or global BOLD signal changes above 5 standard deviations (Nieto-Castanon, 2022; Power et al., 2014), and a reference BOLD image was computed for each subject by averaging all scans excluding outliers. Functional and anatomical data were normalized into standard MNI space, segmented into grey matter, white matter, and CSF tissue classes, and resampled to 2 mm isotropic voxels following a direct normalization procedure (Calhoun et al., 2017; Nieto-Castanon, 2022) using SPM unified segmentation and normalization algorithm (Ashburner, 2007; Ashburner & Friston, 2005) with the default IXI-549 tissue probability map template. Last, functional data were smoothed using spatial convolution with a Gaussian kernel of 8 mm full width half maximum (FWHM).

**Denoising:** In addition, functional data were denoised using a standard denoising pipeline (Nieto-Castanon, 2020) including the regression of potential confounding effects characterized by outliers regressors (10 components), motion parameters and their first order derivatives (12 factors) (Friston, Williams, Howard, Frackowiak, & Turner, 1996), white matter timeseries (16 CompCor noise components), CSF timeseries (16 CompCor noise components), and linear trends (2 factors) within each functional run, followed by bandpass frequency filtering of the BOLD timeseries (Hallquist, Hwang, & Luna, 2013) between 0.01 Hz and 0.1 Hz. CompCor (Behzadi, Restom, Liau, & Liu, 2007; Chai, Castañón, Öngür, & Whitfield-Gabrieli, 2012) noise components within white matter and CSF were estimated by computing the average BOLD signal as well as the largest principal components orthogonal to the BOLD average, motion parameters within each subject's eroded segmentation masks.

**First-level analysis:** Seed-based connectivity maps (SBC) were estimated characterizing the spatial pattern of functional connectivity with a seed area while controlling for all other seeds. Seed regions included the left dlPFC (-43,33,28), left PPC (-46,-58,49), right dlPFC, (41,38,30) and right PPC (52,-52,45). Functional connectivity strength was represented by multivariate regression coefficients from a weighted general linear model (Nieto-Castanon, 2020), estimated separately for each target voxel, modeling the association between all seeds simultaneously and each individual voxel BOLD signal timeseries. Individual scans were weighted by a boxcar signal characterizing each experimental condition (0-Back; 2-Back) convolved with an SPM canonical hemodynamic response function and rectified.

**Group-level analyses:** A General Linear Model (GLM) was conducted for the group-level analyses (Nieto-Castanon, 2020). For each individual voxel a separate GLM was estimated, with first-level connectivity measures at this voxel as dependent variables (one independent sample per subject and one measurement per experimental condition (2-Back; 0-Back)), and groups (high vs. low CTQ; patients vs. controls) as independent variables. Voxel-level hypotheses were evaluated using multivariate parametric statistics with random-effects across subjects and sample covariance estimation across multiple measurements. Inferences were performed at the level of individual clusters (groups of contiguous voxels). Cluster-level inferences were based on parametric statistics from Gaussian Random Field theory (Nieto-Castanon, 2020; Worsley et al., 1996). Results were thresholded using a combination of a cluster-forming p < 0.001 voxel-level threshold, and a familywise corrected p-FDR < 0.05 cluster-size threshold (Chumbley, Worsley, Flandin, & Friston, 2010).

**Supplementary Table 2A.**

*Means and Group Differences for Mean Framewise Displacement and Outlier Count for Patients and Healthy Participants*

|  | **Patients (*n* = 36)** | **Healthy Participants (*n* = 124)** | **Statistics** | |
| --- | --- | --- | --- | --- |
|  | **Mean (*SD*)/Median (IQR)** | **Mean (*SD*)/Median (IQR)** | ***t*_(39.35)_ / *W*** | ***p*** |
| **Mean FD** | 0.261 (0.146) | 0.160 (0.067) | 3.854 | <0.001*** |
| **Outlier Count** | 2 (7.25) | 0 (2) | 1581 | 0.003** |

*Note.* Mean FD = mean framewise displacement; SD = standard deviation; IQR = interquartile range. Missing: *N* = 6; patients, *n* = 2; healthy participants, *n* = 4.

**p*<0.05, ***p*<0.01, ****p*<0.001

**Supplementary Table 2B.**

*Means and Group Differences for Mean Framewise Displacement and Outlier Count for Low- and High-Trauma Groups*

|  | **Low-trauma group (*n* = 103)** | **High-trauma group (*n* = 57)** | **Statistics** | |
| --- | --- | --- | --- | --- |
|  | **Mean (SD)/Median (IQR)** | **Mean (SD)/Median (IQR)** | ***t*_(121.93)_ */ W*** | ***p*** |
| **Mean FD** | 0.173 (0.101) | 0.197 (0.095) | -1.533 | 0.128 |
| **Outlier Count** | 0 (2) | 2 (5) | 2237.5 | 0.006** |

*Note.* Assignment to high-vs.-low CTQ groups was based on a median split of total childhood trauma scores; Mean FD = mean framewise displacement; SD = standard deviation; IQR = interquartile range. Missing: *N* = 6; low-trauma group, *n* = 6; high-trauma group, *n* = 0.

**p*<0.05, ***p*<0.01

**Supplementary Table 3.**

*Means and Group Differences for Sex, Age and Years of Education for Low- and High-Trauma Groups*

| **Characteristics** | **Low-Trauma group (*N* = 57)** | **High-Trauma Group (*N* = 128)** | **Statistics** | |
| --- | --- | --- | --- | --- |
|  | **Mean (SD)** | **Mean (SD)** | **t/χ2/W** | ***p*** |
| Gender (male:female) | 66:43 | 37:20 | 0.146 | 0.703 |
| Age (years) | 35.0 (11.7) | 40.3 (12.1) | -2.74 | 0.007** |
| Years of education^a^ | 16.7 (3.62) | 15.8 (4.0) | 3479.5 | 0.203 |

*Note.* Low- and high-trauma groups were assigned using a mean split for total CTQ score; chi-square test was used to compare frequencies of males and females across trauma groups; independent samples t-test was used to compare age across trauma groups; Wilcoxon signed ranks test was used to compare years of education (post-primary) across trauma groups

^a^Missing data for years of education: *N* = 6

**p*<0.05 ***p*<0.01

**Supplementary Table 4**

*Regression Results for Childhood Trauma Questionnaire (CTQ) Total and Subscale Scores and a) Letter-Number Sequencing (LNS) Scores and b) Spatial Working Memory Total Errors (SWMTE)*

| **Predictor** | **Outcome** | ***B*** | ***SE*** | ***β*** | **t** | ***p*** | **adjusted *p*** |
| --- | --- | --- | --- | --- | --- | --- | --- |
| Total CTQ | LNS | -0.0312 | 0.0165 | -0.1442 | -1.896 | 0.0598 | 0.3589 |
| Emotional abuse | LNS | -0.0764 | 0.0487 | -0.1184 | -1.57 | 0.1185 | 0.7109 |
| Emotional neglect | LNS | -0.0088 | 0.0498 | -0.0135 | -0.176 | 0.8601 | 1 |
| Physical abuse | LNS | -0.1758 | 0.0706 | -0.1855 | -2.49 | 0.0138* | 0.0827 |
| Physical neglect | LNS | -0.2595 | 0.0824 | -0.2342 | -3.151 | 0.0019** | 0.0116* |
| Sexual abuse | LNS | -0.0438 | 0.0769 | -0.0440 | -0.57 | 0.5698 | 1 |
| Total CTQ | SWMTE | 0.0893 | 0.0489 | 0.1299 | 1.827 | 0.0695 | 0.4172 |
| Emotional abuse | SWMTE | 0.1384 | 0.1452 | 0.0674 | 0.954 | 0.3417 | 1 |
| Emotional neglect | SWMTE | 0.2811 | 0.1461 | 0.1361 | 1.924 | 0.0562 | 0.337 |
| Physical abuse | SWMTE | 0.3723 | 0.2116 | 0.1235 | 1.76 | 0.0804 | 0.4823 |
| Physical neglect | SWMTE | 0.5045 | 0.2488 | 0.1432 | 2.028 | 0.0442* | 0.2652 |
| Sexual abuse | SWMTE | 0.0580 | 0.2286 | 0.0183 | 0.254 | 0.7999 | 1 |

*Note.* Sex, age, and education history (years of post-primary education) were included as covariates. Bonferroni correction was applied to adjust for multiple comparisons. *B* = unstandardized regression coefficient; *SE* = standard error of the coefficient; *β* = standardised regression coefficient; *t* = t statistic; *p* = uncorrected *p*-value; adjusted *p* = adjusted *p*-value after Bonferroni correction; LNS = Letter-Number Sequencing total score; SWMTE = Spatial Working Memory Total Errors.

**p*<0.05 ***p*<0.01

**Supplementary Figure 1.**

Scatterplots showing the associations between childhood trauma (CT) scores (total CTQ and all 5 subscales) and working memory performance as measured by the letter-number sequencing (LNS) test. The panels below show the associations between: **(a)** physical neglect and LNS scores (*B* = -0.26, *p* = 0.0019**, *p*-adj = 0.0116*), **(b)** emotional neglect and LNS scores (*B* = -0.01, *p* = 0.8601, *p*-adj = 1), **(c)** physical abuse and LNS scores (*B* = -0.18, *p* = 0.0138*, *p*-adj = 0.0827), **(d)** emotional abuse and LNS scores (*B* = -0.08, *p* = 0.1185, *p*-adj = 0.7109), **(e)** sexual abuse and LNS scores (*B* = -0.04, *p* = 0.0695, *p*-adj = 1), **(f)** total CT and LNS scores (*B* = -0.03, *p* = 0.0598, *p*-adj = 0.3589).

**Supplementary Figure 2.**

*Scatterplots showing the associations between childhood trauma (CT) scores (total CTQ and all 5 subscales) and working memory performance as measured by the spatial working memory total errors (SWMTE).*

The panels below show the associations between: **(a)** physical neglect and SWMTE (*B* = 0.50, *p* = 0.0442*, *p*-adj = 0.2652), **(b)** emotional neglect and SWMTE (*B* = 0.2811, *p* = 0.0562, *p*-adj = 0.337), **(c)** physical abuse and SWMTE (*B* = 0.37, *p* = 0.0804, *p*-adj = 0.4823), **(d)** emotional abuse and SWMTE (*B* = 0.14, *p* = 0.3417, *p*-adj = 1), **(e)** sexual abuse and SWMTE (*B* = 0.06, *p* = 0.7999, *p*-adj = 1), **(f)** total CT and SWMTE (*B* = 0.0893, *p* = 0.0695, *p*-adj = 0.4172).

**Supplementary Table 5.**

*Significant Seed-Based Associations between Frontoparietal Network Seed Regions and Target Voxels Brain-Wide for the 2-Back > 0-Back Contrast Across the Whole Sample*

| **Seed Region** | **MNI space (x, y, z)** | **Target Region** | **MNI space (x, y, z)** | **Voxels** | **Peak T** | **Height Threshold *p*-FDR value** |
| --- | --- | --- | --- | --- | --- | --- |
| left dlPFC | (-43, 33, 28) | Frontal Medial Cortex | (-02, 48, -12) | 2206 | -5.4010 | <0.0001*** |
| left dlPFC | (-43, 33, 28) | left Lingual Gyrus | (-16, -38, -06) | 2127 | -5.5977 | <0.0001*** |
| left dlPFC | (-43, 33, 28) | Lateral Occipital Cortex^a^ | (10, -58, 68) | 621 | 4.7783 | <0.0001*** |
| left dlPFC | (-43, 33, 28) | right Superior Parietal Lobule | (46, -42, 58) | 538 | 5.0557 | <0.0001*** |
| left dlPFC | (-43, 33, 28) | right Superior Frontal Gyrus | (28, 06, 66) | 447 | 5.3573 | <0.0001*** |
| left dlPFC | (-43, 33, 28) | Lateral Occipital Cortex^b^ | (-54, -64, 36) | 424 | -4.9685 | <0.0001*** |
| left dlPFC | (-43, 33, 28) | Lateral Occipital Cortex^a^ | (42, -90, -04) | 301 | -4.5177 | 0.0025** |
| left dlPFC | (-43, 33, 28) | left Middle Temporal Gyrus | (-60, -12, -16) | 271 | -4.9015 | 0.0037** |
| left dlPFC | (-43, 33, 28) | right Frontal Pole | (40, 40, 28) | 258 | 4.5231 | 0.0042** |
| left dlPFC | (-43, 33, 28) | left Superior Parietal Lobule | (-46, -40, 52) | 244 | 4.2035 | 0.0050** |
| left dlPFC | (-43, 33, 28) | left Cerebellum (7b) | (-38, -46, -40) | 234 | 4.2715 | 0.0055** |
| left dlPFC | (-43, 33, 28) | right Central Opercular Cortex | (50, -08, 06) | 180 | -4.5802 | 0.0151* |
| left dlPFC | (-43, 33, 28) | left Frontal Orbital Cortex | (-22, 10, -16) | 131 | -5.2018 | 0.0419* |
| right dlPFC | (41, 38, 30) | posterior Middle Temporal Gyrus, right | (64, -24, -10) | 501 | -4.7248 | 0.0004*** |

*Note.* dlPFC = dorsolateral prefrontal cortex; PPC = posterior parietal cortex; ^a^superior division right, ^b^superior division left, ^c^posterior division right

**p*<0.05 ***p*<0.01 ****p*<0.001

**Supplementary Figure 3.**

*Heat Maps of Seed-based Connectivity (SBC) Results for Frontoparietal Seed Regions Showing Significant Differences in Connectivity under Increasing Working Memory (WM) Load (2-Back > 0-Back contrast).*

A. Left dorsolateral prefrontal cortex (dlPFC) seed: thirteen clusters showed significantly increased (yellow-red) or decreased (green-blue) connectivity under increasing WM load.
B. Right dlPFC seed: one cluster showed significantly reduced connectivity under increasing WM load.

**Supplementary Table 6.**

*Significant Seed-Based Associations Showing Group Differences for Controls > Patients between Frontoparietal Network Seed Regions and Target Voxels Brain-Wide during the 0-Back Condition*

| **Seed Region** | **MNI space**  **(x, y, z)** | **Target Region** | **MNI space**  **(x, y, z)** | **Voxels** | **Peak T** | **Height Threshold PFDR value** | |
| --- | --- | --- | --- | --- | --- | --- | --- |
| left dlPFC | (-43, 33, 28) | Middle Frontal Gyrus Right | (48, 26, 26) | 2231 | 5.4584 | <0.0001*** | |
| left dlPFC | (-43, 33, 28) | Middle Frontal Gyrus Left | (-50, 20, 32) | 1324 | 4.8842 | <0.0001*** | |
| left dlPFC | (-43, 33, 28) | Lateral Occipital Cortex, superior division left | (-34, -64, 42) | 583 | 4.7061 | <0.0001*** | |
| left dlPFC | (-43, 33, 28) | Frontal Pole Left | (-40, 56, -06) | 494 | 5.0592 | 0.0002*** | |
| left dlPFC | (-43, 33, 28) | Angular Gyrus Right | (50, -48, 58) | 281 | 4.5563 | 0.0041** | |
| left dlPFC | (-43, 33, 28) | Cerebellum Crus2 Right | (06, -84, -24) | 234 | 4.2399 | 0.0081** | |
| left dlPFC | (-43, 33, 28) | Cerebellum 7b Right | (36, -70, -54) | 145 | 4.7056 | 0.0422* | |
| right dlPFC | (41, 38, 30) | Middle Frontal Gyrus Right | (42, 34, 26) | 4677 | 6.6822 | <0.0001*** | |
| right dlPFC | (41, 38, 30) | Cerebellum 7b Right | (34, -72, -54) | 3523 | 6.0613 | <0.0001*** | |
| right dlPFC | (41, 38, 30) | Supramarginal Gyrus, posterior division right | (48, -46, 48) | 1793 | 4.9684 | <0.0001*** | |
| right dlPFC | (41, 38, 30) | Frontal Medial Cortex | (-06, 50, -14) | 790 | -5.5918 | <0.0001*** | |
| right dlPFC | (41, 38, 30) | Frontal Pole Left | (-14, 52, 34) | 533 | -4.3186 | <0.0001*** | |
| right dlPFC | (41, 38, 30) | Angular Gyrus Left | (-42, -52, 46) | 251 | 4.6632 | <0.0001*** | |
| right dlPFC | (41, 38, 30) | Inferior Temporal Gyrus, temporooccipital part right | (56, -54, -10) | 240 | 4.9884 | 0.0066** | |
| right dlPFC | (41, 38, 30) | Insular Cortex Right | (30, 22, -02) | 177 | 4.2518 | 0.0193* |  |
| right dlPFC | (41, 38, 30) | Precentral Gyrus Right | (44, -22, 68) | 147 | -5.1973 | 0.0322* |  |
| left PPC | (-46, -58, 49) | Angular Gyrus Left | (-54, -58, 46) | 1679 | 5.9608 | <0.0001*** |  |
| left PPC | (-46, -58, 49) | Middle Frontal Gyrus Right | (48, 18, 40) | 893 | 5.0107 | <0.0001*** |  |
| left PPC | (-46, -58, 49) | Lateral Occipital Cortex, superior division right | (46, -58, 50) | 751 | 5.0842 | <0.0001*** |  |
| left PPC | (-46, -58, 49) | Superior Frontal Gyrus Left | (-02, 30, 46) | 225 | 3.9358 | 0.0161* |  |
| right PPC | (52, -52, 45) | Supramarginal Gyrus, posterior division right | (58, -40, 46) | 2267 | 6.5606 | <0.0001*** |  |
| right PPC | (52, -52, 45) | Frontal Pole Right | (44, 38, 26) | 2007 | 5.2025 | <0.0001*** |  |
| right PPC | (52, -52, 45) | Frontal Pole Left | (-08, 54, 36) | 870 | -4.9219 | <0.0001*** |  |

*Note.* dlPFC = dorsolateral prefrontal cortex; PPC = posterior parietal cortex

**p*<.05 ***p*<.01 ****p*<.001

**Supplementary Figure 4.**

*Heat Maps of Seed-Based Connectivity (SBC) Results for Frontoparietal Seed Regions Showing Significant Differences in Connectivity Between Healthy Participants and Individuals with Schizophrenia During the 0-Back Condition*

A. Right dorsolateral prefrontal cortex (dlPFC) seed: seven clusters showed significantly increased (yellow-red) or decreased (green-blue) connectivity in SZ when compared to healthy participants.
B. Left dlPFC seed: nine clusters showed significantly increased or decreased connectivity in SZ compared to healthy participants.
C. Right posterior parietal cortex (PPC) seed: four clusters showed significantly increased or decreased connectivity in SZ compared to healthy participants.
D. Left PPC seed: three clusters showed significantly increased or decreased connectivity in SZ compared to healthy participants.

**Supplementary Table 7.**

*Regression Results for Physical Neglect and* *the Seed-Based Connectivity Measures Between a) the Right dlPFC and Right pMTG, b) Left dlPFC and MedFC, c) Left dlPFC and Left LG*

| **Predictor** | **Outcome** | **B** | ***SE*** | **t** | **p** | **adjusted *p*** |
| --- | --- | --- | --- | --- | --- | --- |
| Physical Neglect | right dlPFC and right pMTG | 0.0019 | 0.0045 | 0.43 | 0.6675 | 1 |
| Physical Neglect | left dlPFC and MedFC | 0.0015 | 0.0051 | 0.304 | 0.7619 | 1 |
| Physical Neglect | left dlPFC and left LG | -0.0036 | 0.004 | -0.902 | 0.3685 | 1 |

*Note.* Sex, age, and education history (years of post-primary education) were included as covariates. Bonferroni correction was applied to adjust for multiple comparisons.

B = unstandardized regression coefficient; *SE* = standard error of the coefficient; t = t statistic; *p* = uncorrected p-value; adjusted *p* = adjusted p-value after Bonferroni correction; dlPFC = dorsolateral prefrontal cortex; pMTG = Middle Temporal Gyrus, posterior division; MedFC = Frontal Medial Cortex; left LG = left Lingual Gyrus.

**Supplementary Figure 5.**

*Scatterplots Showing the Associations Between Physical Neglect (PN) Scores and Frontoparietal Connectivity. The Panels Below Show the Associations Between:* ***(a)*** *PN and Connectivity Between the Left dorsolateral Prefrontal Cortex (dlPFC) and Frontal Medial Cortex (MedFC) (B = 0.0015, p = 0.7619, p-adj = 1),* ***(b)*** *PN and Connectivity Between the Right dlPFC and the Middle Temporal Gyrus, posterior division (pMTG) (B = 0.0019, p = 0.6675, p-adj = 1),* ***(c)*** *PN and Connectivity Between the Left dlPFC and Left Lingual Gyrus (LG) (B = -0.0036, p = 0.3685, p-adj = 1).*

**Supplementary Table 8.**

*Regression Results for the seed-based connectivity measures between a) the right dlPFC and right pMTG, b) left dlPFC and MedFC, c) left dlPFC and left LG, and letter-number sequencing scores*

| **Predictor** | **Outcome** | **B** | ***SE*** | **t** | ***p*** | **adjusted *p*** |
| --- | --- | --- | --- | --- | --- | --- |
| right dlPFC and right pMTG | LNS | -1.624 | 1.495 | -1.086 | 0.2791 | 0.8372 |
| left dlPFC and MedFC | LNS | -3.22 | 1.294 | -2.488 | 0.0139* | 0.0416* |
| left dlPFC and left LG | LNS | -2.746 | 1.672 | -1.642 | 0.1025 | 0.3076 |

*Note.* Sex, age, and education history (years of post-primary education) were included as covariates. Bonferroni correction was applied to adjust for multiple comparisons.

B = unstandardized regression coefficient; *SE* = standard error of the coefficient; t = t statistic; *p* = uncorrected p-value; adjusted *p* = adjusted p-value after Bonferroni correction; LNS = letter-number sequencing; dlPFC = dorsolateral prefrontal cortex; pMTG = Middle Temporal Gyrus, posterior division; MedFC = Frontal Medial Cortex; left LG = left Lingual Gyrus.

**p*<0.05

**Supplementary Figure 6.**

*Scatterplots Showing the Associations Between Frontoparietal Connectivity and Letter Number Sequency (LNS) Scores*

The Panels Below Show the Associations Between: **(a)** Connectivity of the Left dorsolateral Prefrontal Cortex (dlPFC) and Frontal Medial Cortex (MedFC) and LNS Scores (*B* = -3.22, *p* = 0.0139*, *p*-adj = 0.0416*), **(b)** Connectivity of the Right dlPFC and the Middle Temporal Gyrus, posterior division (pMTG) and LNS Scores (*B* = -1.624, *p* = 0.2791, *p*-adj = 0.8372), **(c)** Connectivity of the Left dlPFC and Left Lingual Gyrus (LG) (*B* = -2.746, *p* = 0.1025, *p*-adj = 0.3076).

**Supplementary Table 9.**

*Moderated Mediation (PROCESS Macro Model 59) Results for the Model with Connectivity between the Left Dorsolateral Prefrontal Cortex and the Frontal Medial Cortex (MedFC) as the Mediator*

| **Variable** | **Model a-path** | | **Model b/c’-path** | |
| --- | --- | --- | --- | --- |
|  | b | *p* | b | *p* |
| PN | -0.0096 | 0.1326 | -0.0794 | 0.3993 |
| Diagnosis | -0.0828 | 0.2967 | -2.5715 | 0.0538 |
| PN x Diagnosis | 0.0237 | 0.0223* | -0.1297 | 0.4256 |
| FC |  |  | -0.5404 | 0.659 |
| FC x Diagnosis |  |  | -10.293 | 0.0112* |

*Note.* There was evidence for a moderated mediation effect as indicated by the index of moderated mediation (*index* = -0.1582, BootSE = 0.1064, 95% CI: [-0.4141, -0.008]). This effect was driven by the moderation of the a-path (*R²* change = 2.98%, *p* = 0.0223) and b-path (*R²* change = 2.75%, *p* = 0.0112) by Diagnosis

PN = physical neglect, FC = functional connectivity between the left dorsolateral Prefontal Cortex and the Frontal Medial Cortex

**p*<0.05

**Supplementary Table 10.**

*Zero-Order Correlation Matrix Between Childhood Trauma Scores (All 5 Subscales and Total Trauma Scores), Working Memory Performance (Letter-Number Sequencing Scores, and Spatial Working Memory Total Errors), and Frontoparietal Connectivity (Left dorsolateral Prefrontal Cortex (dlPFC) and Frontal Medial Cortex (MedFC); Left dlPFC and Left Lingual Gyrus (LG); Right dlPFC and the Middle Temporal Gyrus, posterior division (pMTG))*

|  | Physical Abuse | Sexual Abuse | Emotional Neglect | Physical Neglect | Total Trauma | LNS | SWMTE | left dlPFC-MedFC | left dlPFC-left LG | right dlPFC-pMTG |
| --- | --- | --- | --- | --- | --- | --- | --- | --- | --- | --- |
| Emotional Abuse | 0.66*** | 0.28*** | 0.67*** | 0.65*** | 0.90*** | -0.14 | 0.11 | 0.12 | -0.01 | -0.02 |
| Physical Abuse |  | 0.11 | 0.56*** | 0.58*** | 0.77*** | -0.21** | 0.16* | 0.15 | -0.01 | 0.17* |
| Sexual Abuse |  |  | 0.15 | 0.12 | 0.41*** | -0.08 | 0.11 | 0.13 | -0.04 | 0.03 |
| Emotional Neglect |  |  |  | 0.65*** | 0.84*** | -0.05 | 0.20** | 0.15 | 0 | 0.07 |
| Physical Neglect |  |  |  |  | 0.79*** | -0.27*** | 0.21** | 0.05 | -0.05 | 0.04 |
| Total Trauma |  |  |  |  |  | -0.18* | 0.21** | 0.16* | -0.03 | 0.07 |
| LNS |  |  |  |  |  |  | -0.44*** | -0.23** | -0.18* | -0.11 |
| SWMTE |  |  |  |  |  |  |  | 0.13 | 0.06 | 0.03 |
| left dlPFC-MedFC |  |  |  |  |  |  |  |  | 0.56*** | 0.21** |
| left dlPFC-left LG |  |  |  |  |  |  |  |  |  | 0.24** |

*Note.* Values stated are Pearson’s r values; *N* = 166

**p*<0.05, ***p*<0.01, ****p*<0.001
